# Mitochondrial S‐adenosylmethionine deficiency induces mitochondrial unfolded protein response and extends lifespan in *Caenorhabditis elegans*

**DOI:** 10.1111/acel.14103

**Published:** 2024-02-15

**Authors:** Tse Yu Chen, Feng‐Yung Wang, Pin‐Jung Lee, Ao‐Lin Hsu, Tsui‐Ting Ching

**Affiliations:** ^1^ Institute of Biopharmaceutical Sciences National Yang Ming Chiao Tung University Taipei Taiwan; ^2^ Institute of Biochemistry and Molecular Biology National Yang Ming Chiao Tung University Taipei Taiwan; ^3^ Department of Biological Science & Technology and Institute of Biochemistry and Molecular Biology China Medical University Taichung Taiwan; ^4^ Department of Internal Medicine, Division of Geriatric and Palliative Medicine University of Michigan Medical School Ann Arbor Michigan USA

**Keywords:** longevity, mitochondrial tRNA methyltransferase, S‐adenosyl methionine, UPR^mt^

## Abstract

S‐adenosylmethionine (SAM), generated from methionine and ATP by S‐adenosyl methionine synthetase (SAMS), is the universal methyl group donor required for numerous cellular methylation reactions. In *Caenorhabditis elegans*, silencing *sams‐1*, the major isoform of SAMS, genetically or via dietary restriction induces a robust mitochondrial unfolded protein response (UPR^mt^) and lifespan extension. In this study, we found that depleting SAMS‐1 markedly decreases mitochondrial SAM levels. Moreover, RNAi knockdown of SLC‐25A26, a carrier protein responsible for transporting SAM from the cytoplasm into the mitochondria, significantly lowers the mitochondrial SAM levels and activates UPR^mt^, suggesting that the UPR^mt^ induced by *sams‐1* mutations might result from disrupted mitochondrial SAM homeostasis. Through a genetic screen, we then identified a putative mitochondrial tRNA methyltransferase TRMT‐10C.2 as a major downstream effector of SAMS‐1 to regulate UPR^mt^ and longevity. As disruption of mitochondrial tRNA methylation likely leads to impaired mitochondrial tRNA maturation and consequently reduced mitochondrial translation, our findings suggest that depleting mitochondrial SAM level might trigger UPR^mt^ via attenuating protein translation in the mitochondria. Together, this study has revealed a potential mechanism by which SAMS‐1 regulates UPR^mt^ and longevity.

## Graphical abstract

Mitochondrial SAM deficiency may result in a reduction of mt‐tRNA methylation, leading to the inhibition of protein translation within mitochondria. This, in turn, triggers the mitochondrial unfolded protein response and extends the lifespan.

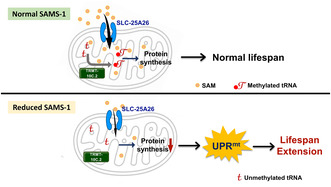

**Abbreviations**
DR: dietary restriction
ELISA: enzyme‐linked immunosorbent assay
FCCP: carbonyl cyanide‐p‐trifluoromethoxyphenylhydrazone
IPTG: isopropyl‐β‐d‐1‐thiogalactopyranoside
KD: knock‐down
MAT1A: methionine adenosyltransferase 1A
MT: methyltransferase
mtRNaseP: mitochondrial RNase P complex
NGM: nematode growth medium
OCR: mitochondrial oxygen consumption rate
RNAi: RNA interference
SAM: S‐adenosyl methionine
SAMS: S‐adenosyl methionine synthetase
UPR^ER^
: endoplasmic reticulum unfolded protein response
UPR^mt^
: mitochondrial unfolded protein response
WT: wild‐type

## 1 INTRODUCTION

S‐adenosylmethionine (SAM) is the ubiquitous methyl donor essential to numerous biological processes. Cellular SAM is generated from methionine and ATP by S‐adenosyl methionine synthetase (SAMS), a highly conserved enzyme throughout evolution (Kotb et al., 1997). In *Caenorhabditis elegans*, *sams‐1* is recognized as the ortholog of mammalian methionine adenosyltransferase 1A (MAT1A). RNAi knockdown of *sams‐1* significantly reduced SAM levels in worms (Walker et al., 2011). Moreover, knockdown or knockout of *sams‐1* markedly extends the lifespan of wild‐type animals (Hansen et al., 2005). Since depletion of *sams‐1* fails to further extend lifespan in *eat‐2* mutants and the lifespan extension mediated by *sams‐1* is independent of *daf‐16*, it is believed that *sams‐1* acts downstream to mediate the dietary restriction (DR)‐induced longevity.

SAM‐dependent methyltransferases (MTs) are enzymes that transfer methyl groups from SAM to their substrates. SAM‐dependent MTs can be found in various organelles, including mitochondria (Petrossian & Clarke, 2011; Rhein et al., 2013). Notably, the substrates of the mitochondrial SAM‐dependent MTs range from DNA, RNA, protein, and small molecules, implicating an essential role of SAM‐dependent methylations in mitochondrial functions. The SAM molecules in the mitochondrial matrix are mainly transported from the cytoplasm through SLC25A26, the only mitochondrial SAM transporter known to date (Agrimi et al., 2004). In *Drosophila* and mice, loss of function mutations of SLC25A26 led to embryonic lethality (Cheng et al., 2022; Schober et al., 2022). Moreover, SLC25A26 mutations in humans resulted in various mitochondrial defects due to reduced intra‐mitochondrial methylation (Kishita et al., 2015), further highlighting the importance of maintaining mitochondrial SAM homeostasis for normal cell functions. However, while the functions and the regulations of many mitochondrial MTs have been well‐studied, how cells control the availability of the methyl group donor SAM in the mitochondria in response to different internal or external cues was largely unexplored. In particular, how animals regulate mitochondrial SAM homeostasis in response to dietary changes in the context of longevity regulation remains unknown.

In *C*. *elegans*, RNAi knockdown of *sams‐1* induced a robust mitochondrial unfolded protein response (UPR^mt^) (Hou et al., 2014). In this study, we found that the *sams‐1*‐mediated UPR^mt^ is mainly caused by SAM deficiency in the mitochondria and that depleting *slc‐25A26* is sufficient to activate UPR^mt^ in *C*. *elegans*. Our findings also suggested that the attenuation in mitochondrial tRNA methylation due to mitochondrial SAM deficiency may potentially account for the elevated UPR^mt^ response and extended lifespan observed in SAM‐deficient animals.

## 2 RESULTS

### 2.1 SLC‐25A26 functions as a mitochondrial SAM transporter in *C*. *Elegans*

**FIGURE 1:**
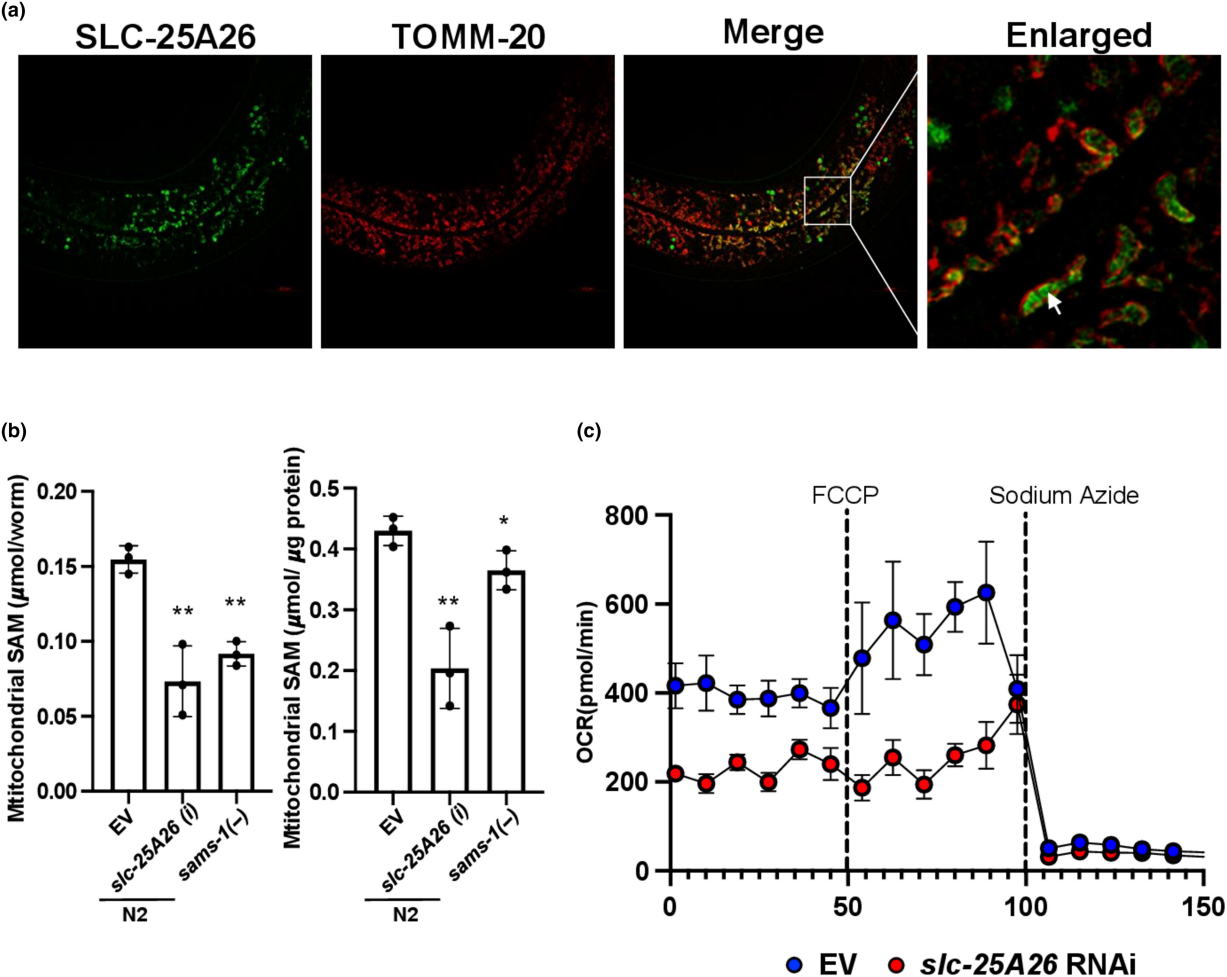
The subcellular distribution and functions of SLC‐25A26 in *C*. *elegans*. (a) Representative images showing the co‐localization of intestinal SLC‐25A26 (green) and TOMM‐20 (red) in worms carrying intestinal SLC‐25A26::GFP and TOMM‐20::mCherry. scale bars = 10 μm. (b) Mitochondrial SAM levels in Day1 adult *sams‐1* mutants and wild‐type worms treated with control vector or *slc‐25A26* RNAi. SAM levels were normalized to either number of worms (left) or protein amount (right). Individual points represent independent experiments. *p < 0.05; **p < 0.01 (Student's *t*‐test) (c) Representative Oxygen Consumption Rate (OCR) of L4 wild‐type worms treated with control vector or *slc‐25A26* RNAi was measured by Seahorse Analysis. Three biological replicates with 30 worms per replicate.

The *C*. *elegans slc‐25A26* gene encodes a potential ortholog of the mammalian *SLC25A26*. To validate that worm SLC‐25A26 indeed functions as a mitochondrial SAM transporter, we first generated animals carrying *slc‐25A26p*::*GFP* transgene to examine the expression pattern of SLC‐25A26. We found that SLC‐25A26 is mainly expressed in the pharynx, intestine, and excretory canal cells (Figure S1a). We then generated transgenic animals expressing both SLC‐25A26::GFP and TOMM‐20::mCherry, a mitochondrial outer membrane marker, in the intestinal cells to determine the subcellular distribution of SLC‐25A26 in the cells. Indeed, we found that SLC‐25A26::GFP signals largely overlapped with TOMM‐20::mCherry signals (Figure 1a). Furthermore, SLC‐25A26 seems to be located in the inner membrane of mitochondria (Figure 1a).

Next, we purified mitochondria from wild‐type worms fed with either *sams‐1* or *slc‐25A26* RNAi and then measured the mitochondrial SAM levels by ELISA. Since SAMS‐1 is the primary enzyme that generates SAM in worms (Walker et al., 2011), we anticipated the overall cellular SAM level as well as its level in the mitochondria to be reduced in *sams‐1* mutants. Indeed *sams‐1* mutants significantly reduced mitochondrial SAM levels (Figure 1b). Moreover, knocking down *slc‐25A26* by RNAi reduces mitochondrial SAM levels to a similar extent (Figure 1b), supporting the idea that SLC‐25A26 functions as a mitochondrial SAM transporter in *C*. *elegans*. It is worth noting that while global protein synthesis is greatly reduced in *sams‐1* mutant, it is not affected in *slc‐25A26* RNAi animals. Similarly, RNAi depletion of *slc‐25A26* did not have a significant impact on reproduction and development (Figure S1c–e), as observed in *sams‐1* mutants. We then examined the mitochondria functions in *slc‐25A26* RNAi mutants by measuring the oxygen consumption rate (OCR). RNAi depletion of *slc‐25A26* significantly impaired mitochondrial respiration, as both basal and maximal respiration activities were suppressed in *slc‐25A26* RNAi KD animals (Figure 1c), indicating that lowering SAM supply into the mitochondria might perturb mitochondrial functions.

### 2.2 SAM deficiency in the mitochondria induces UPR^mt^

**FIGURE 2:**
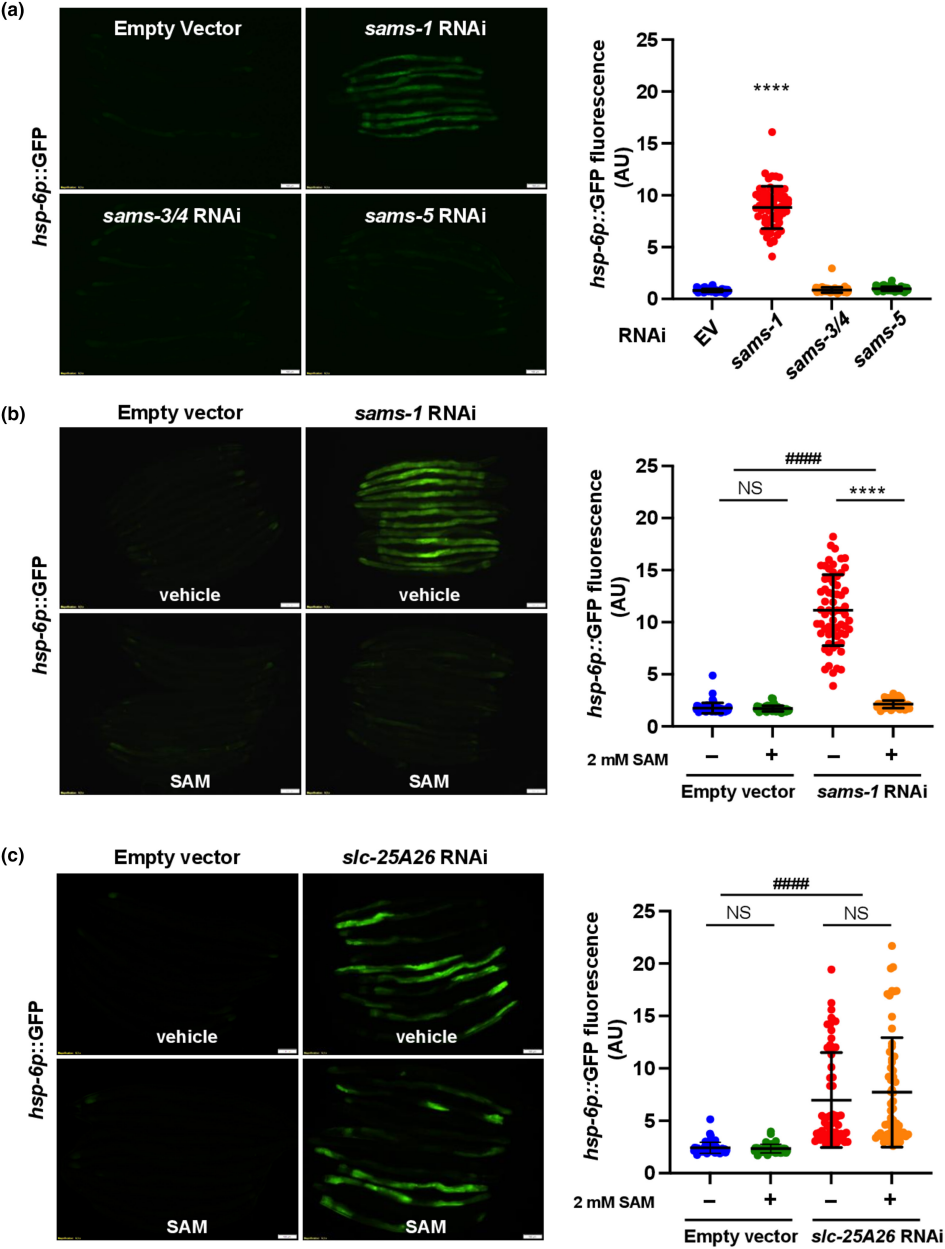
Inhibiting SLC‐25A26‐dependent SAM import into mitochondria activates UPR^mt^. (a) Representative images of *hsp‐6p*::*GFP* expression in Day 1 adult of wild‐type worms grown on either empty vector (EV), *sams‐1*, *sams‐3/4,* or *sams‐5* RNAi bacteria (left). Quantitative analysis of *hsp‐6p*::*GFP* expression (right). The central line and error bars indicate the mean and standard deviation, respectively. Levels of significance are shown as ****p < 0.0001 in one‐way ANOVA test. (b, c) Representative images of *hsp‐6p*::*GFP* expression in Day 1 adult worms with indicated genotypes and treatments (left). F_1_ progenies of *hsp‐6p*::*GFP* transgenic worms grown on either empty vector (EV), *sams‐1* RNAi (b), or *slc‐25A26* RNAi (c) bacteria were placed on UV‐killed OP50 bacteria supplemented with vehicle or 2 mM SAM. Quantitative analysis of *hsp‐6p*::*GFP* expression (right). Central line and error bars indicate the mean and standard deviation, respectively. Data were analyzed using two‐way ANOVA with Tukey's multiple comparison test. Levels of significance are shown as ^####^ *p* < 0.0001 (interaction); ****p < 0.0001 (pairwise); NS, not significant. Scale bar = 100 μm. Data are representative of at least two biological replicates with 50 worms for each treatment (*N* ≥ 2; *n* ≥ 50).

Previous research has indicated that reducing SAMS‐1 markedly triggers UPR^mt^ in wild‐type animals (Hou et al., 2014). We thus examined whether other SAMS paralogs are involved in UPR^mt^ regulation. Intriguingly, the activation of UPR^mt^ occurred exclusively through the inhibition of SAMS‐1, with no similar effect observed for other SAMSs (Figure 2a). We have also confirmed that the RNAi clone targeting sams‐1 did not affect the expression of other SAMS paralogs (Figure S1f), eliminating the possibility of off‐target effects (Chen et al., 2020). This phenomenon could potentially be attributed to variations in tissue distribution and differential protein expression levels of SAMS isozymes (Chen et al., 2020; Godbole et al., 2023). We then asked whether the elevated UPR^mt^ observed in *sams‐1* mutants is caused by the reduced SAM level in mitochondria. Using *hsp‐6p*::*GFP* as the reporter for UPR^mt^, we found that supplementation of 2 mM SAM fully rescued the elevated UPR^mt^ in *sams‐1* knockdown animals (Figure 2b), whereas supplementation of 2 mM methionine or 2 mM homocysteine failed to inhibit *sams‐1* RNAi‐induced UPR^mt^ (Figure S2a). These results implied that reducing SAM levels generated by SAMS‐1 is required for inducing UPR^mt^ in *sams‐1* RNAi mutants. Furthermore, UPR^mt^ is markedly increased by knocking down *slc‐25A26* (Figure 2c), which would prevent SAM from transporting across the mitochondrial membranes and consequently lower the mitochondrial SAM level. As expected, this increased UPR^mt^ induced by *slc‐25A26* RNAi cannot be reversed by SAM supplement (Figure 2c). Since both ATFS‐1 and DVE‐1 are transcription factors crucial for the transcription UPR^mt^‐related gene (Shpilka & Haynes, 2018), we then examined whether *atfs‐1* and *dve‐1* are required for the SAM deficiency‐induced UPR^mt^. Indeed, we found that the UPR^mt^ induced by *slc‐25A26* depletion is significantly suppressed by RNAi knockdown of *atfs‐1* or *dve‐1* (Figure S2b,c). Together, our findings suggest that the UPR^mt^ observed in *sams‐1* mutants might be a result of SAM deficiency in mitochondria and that SAM deficiency in the mitochondria alone is sufficient to induce UPR^mt^ in *C*. *elegans*.

### 2.3 A putative mitochondrial tRNA methyltransferase TRMT‐10C.2 mediates the SAM deficiency‐induced UPR^mt^

**FIGURE 3:**
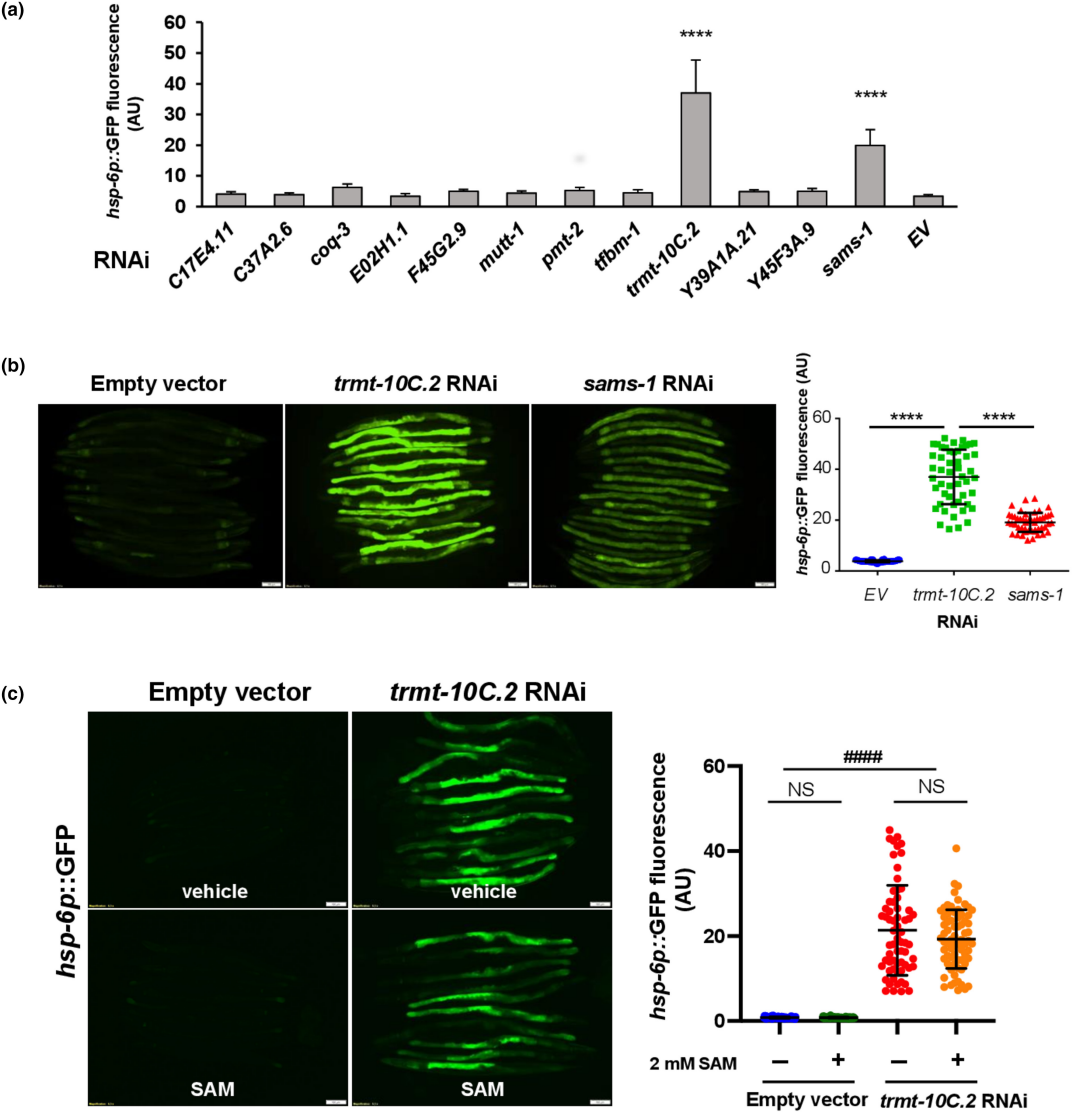
Suppression of mitochondrial TRMT‐10C.2 activity induces UPR^mt^ in *C*. *elegans*. (a) *hsp‐6p*::*GFP* expression was quantified for 11 RNAi clones corresponding to the putative mitochondrial methyltransferases. Results are expressed as mean ± SD for *n* = 50 per treatment. (b) Representative images of *hsp‐6p*::*GFP* expression in wild‐type worms fed with empty vector (EV), *trmt‐10C*.*2*, and *sams‐1* RNAi bacteria. Right panel, quantitative analysis of *hsp‐6p*::*GFP* expression. Scale bar = 100 μm. Data from a and b were analyzed by one‐way ANOVA. Levels of significance were shown as ****p < 0.0001. (c) Day 1 F1 progeny of *hsp‐6p*::*GFP* transgenic worms treated with empty vector (EV) or *trmt‐10C*.*2* RNAi grown on UV‐killed OP50 supplemented with vehicle or 2 mM SAM. Right panels, quantitative analysis of *hsp‐6p*::*GFP* expression. The central line and error bars indicate mean and standard deviation, respectively. Two biological repeats with *n* = 50 per treatment. Data were analyzed by two‐way ANOVA with Tukey's multiple comparison test. Levels of significance are shown as ^####^ *p* < 0.0001 (interaction); NS, not significant (pairwise). Scale bar = 100 μm. Data are representative of at least two biological replicates with 50 worms for each treatment (*N* ≥ 2; *n* ≥ 50).

Since SAM serves as the major methyl group donor for various cellular methylation reactions, we thus asked whether defects in specific methylation(s) in the mitochondria may induce the UPR^mt^ response in *sams‐1* and *slc‐25A26* mutants. To address this, we performed a small‐scale RNAi screen, focusing first on the methyltransferases (MT) predicted to be present in the mitochondria. Among the 11 MT genes we tested, we found that knocking down *trmt‐10C*.*2* (gene sequence name, *C56G2*.*3*), but not the others, could trigger a UPR^mt^ even stronger than knocking down *sams‐1* (Figure 3a,b). *trmt‐10C*.*2* is a homolog of human tRNA methyltransferase 10 homolog C (TRMT10C). TRMT10C is one of the subunits of human mitochondrial RNase P complex (mtRNaseP), which catalyzes purine 9 N1‐methylation of mitochondrial tRNA (Motorin & Helm, 2011; Oerum et al., 2018). Furthermore, the supplementation of additional SAM failed to rescue UPR^mt^ phenotype induced by *trmt‐10C*.*2* depletion (Figure 3c), indicating that TRMT‐10C.2‐catalyzed methylation reaction is a critical downstream effector of SAM deficiency‐induced UPR^mt^.

**FIGURE 4:**
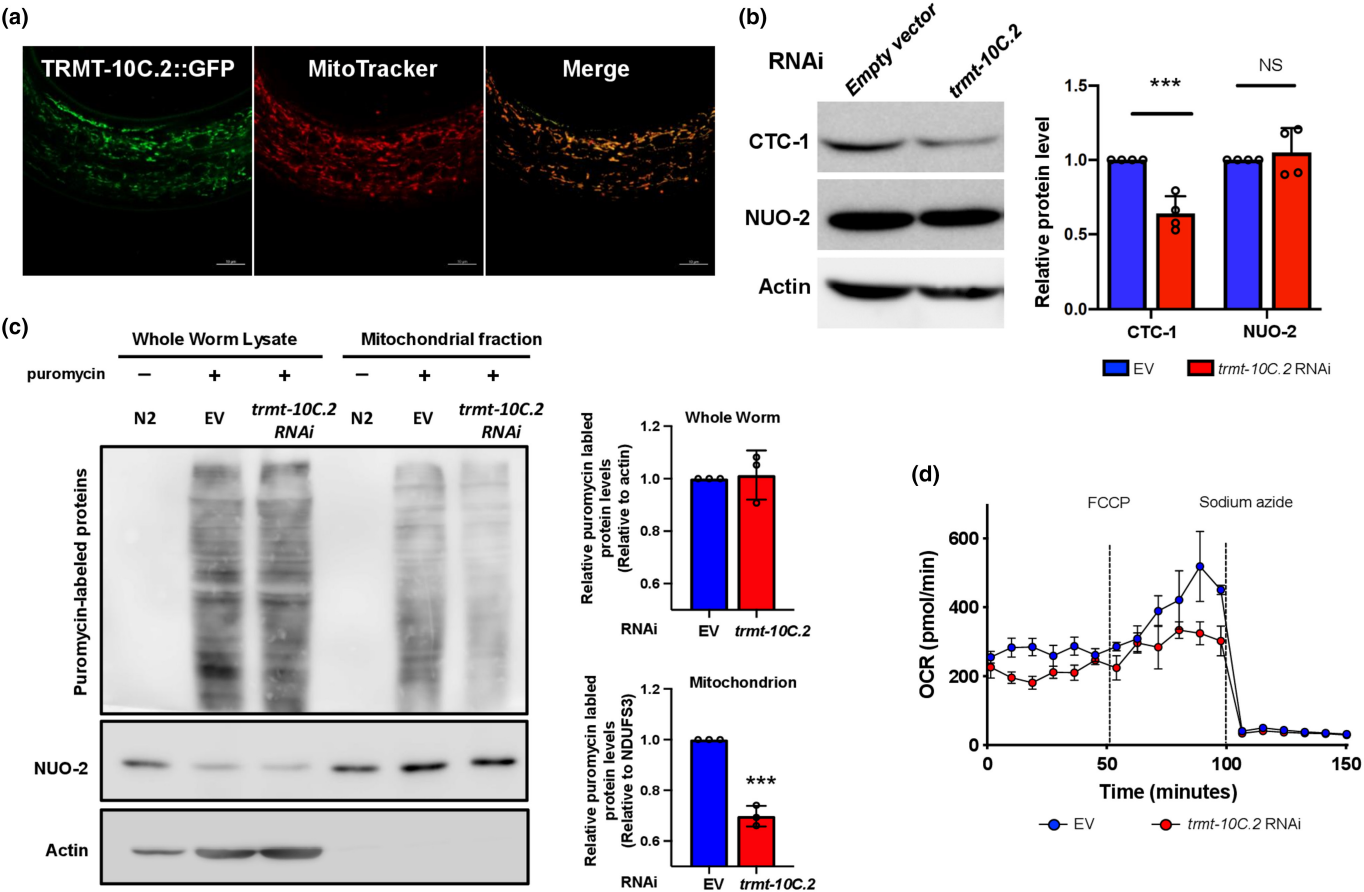
Inactivating TRMT‐10C.2 impairs protein translation and respiration in mitochondria. (a) Representative confocal microscopy images of the co‐localization of TRMT‐10C.2::GFP and MitoTracker Red in L4 transgenic worms carrying *trmt‐10C*.*2*::*GFP*. Scale bar = 10 μm. (b) Representative immunoblots for CTC‐1 and NUO‐2 in wild‐type worms fed with empty vector (EV) and *trmt‐10C*.*2* RNAi bacteria (left). Densitometric quantification of CTC‐1 or NUO‐2: β‐actin ratio relative to EV groups (right). Means ± SEMs for four biological replicates. ***p < 0.001, Student's *t*‐test. (c) Puromycin incorporation followed by western blot analysis using antibodies detecting puromycin, NUO‐2, and actin in the whole worm lysate and mitochondrial lysate of day 1 wild‐type worms treated with empty vector or *trmt‐10C*.*2* RNAi. Densitometric quantification of puromycin incorporation in the whole worm lysate and mitochondrial lysate (right). Means ± SEMs for three biological replicates. ***p < 0.001, Student's *t*‐test. (d) Oxygen Consumption Rate (OCR) of L4 wild‐type worms treated with empty vector or *tmt‐10C*.*2* RNAi was measured by Seahorse Analysis. Data are representative of *n* = 3 biological replicates with 30 worms per treatment.

Next, we created a transgenic animal carrying *trmt‐10C*.*2p*:: *trmt‐10C*.*2*::*GFP* to validate the subcellular localization of TRMT‐10C.2. We found that *trmt‐10C*.*2* is expressed mainly in the intestine (Figure S3a). Moreover, colocalization of TRMT‐10C.2::GFP and mitochondrial marker MitoTracker Red indicates that TRMT‐10C.2 is a mitochondrial protein (Figure 4a). Similar to *slc‐25A26* RNAi treatment, knockdown of *trmt‐10C*.*2* did not exhibit a substantial influence on reproduction and development (Figure S3b–d). mtRNaseP is responsible for removing the 5′ ends of precursor tRNAs during tRNA maturation in human mitochondria (Sanchez et al., 2011). Therefore, when mitochondrial tRNA maturation is attenuated, protein translation in the mitochondria is disturbed. To examine whether the mitochondrial translation is inhibited by depleting *trmt‐10C*.*2*, we measured the protein levels of CTC‐1 and NUO‐2 in wild‐type animals and *trmt‐10C*.*2* RNAi mutants by western blotting. *ctc‐1* is the cytochrome c oxidase subunit 1 encoded by mitochondrial DNA, whereas *nuo‐2* is a nuclear gene encoding a subunit mitochondrial NADH ubiquinone oxidoreductase. Since CTC‐1 and NUO‐2 are translated via two distinct machineries, CTC‐1 and NUO‐2 protein levels are ideal surrogates for protein translation occurring in mitochondria and cytoplasm respectively. Our results showed that RNAi knockdown of *trmt‐10C*.*2* reduces CTC‐1 but not NUO‐2 levels (Figure 4b), suggesting that only mitochondrial translation is affected by *trmt‐10C*.*2* depletion. To further confirm this phenomenon, we also performed puromycin incorporation assay to assess the protein translation rate in both the cytosol and the mitochondria. Worms with *trmt‐10C*.*2* knocked‐down were first labeled with puromycin. Subsequently, whole worm lysate or mitochondrial lysate was prepared for detecting puromycin incorporation through immunoblotting. Likewise, knockdown of *trmt‐10C*.*2* significantly reduced mitochondrial protein synthesis (Figure 4c). As a previous study has shown that mutations in human TRMT10C cause multiple respiratory chain deficiencies (Metodiev et al., 2016), we also examined the OCR in *trmt‐10C*.*2* RNAi mutants. In line with previous findings, RNAi depletion of *trmt‐10C*.*2* significantly lowered mitochondrial respiration in *C*. *elegans* (Figure 4d).

**FIGURE 5:**
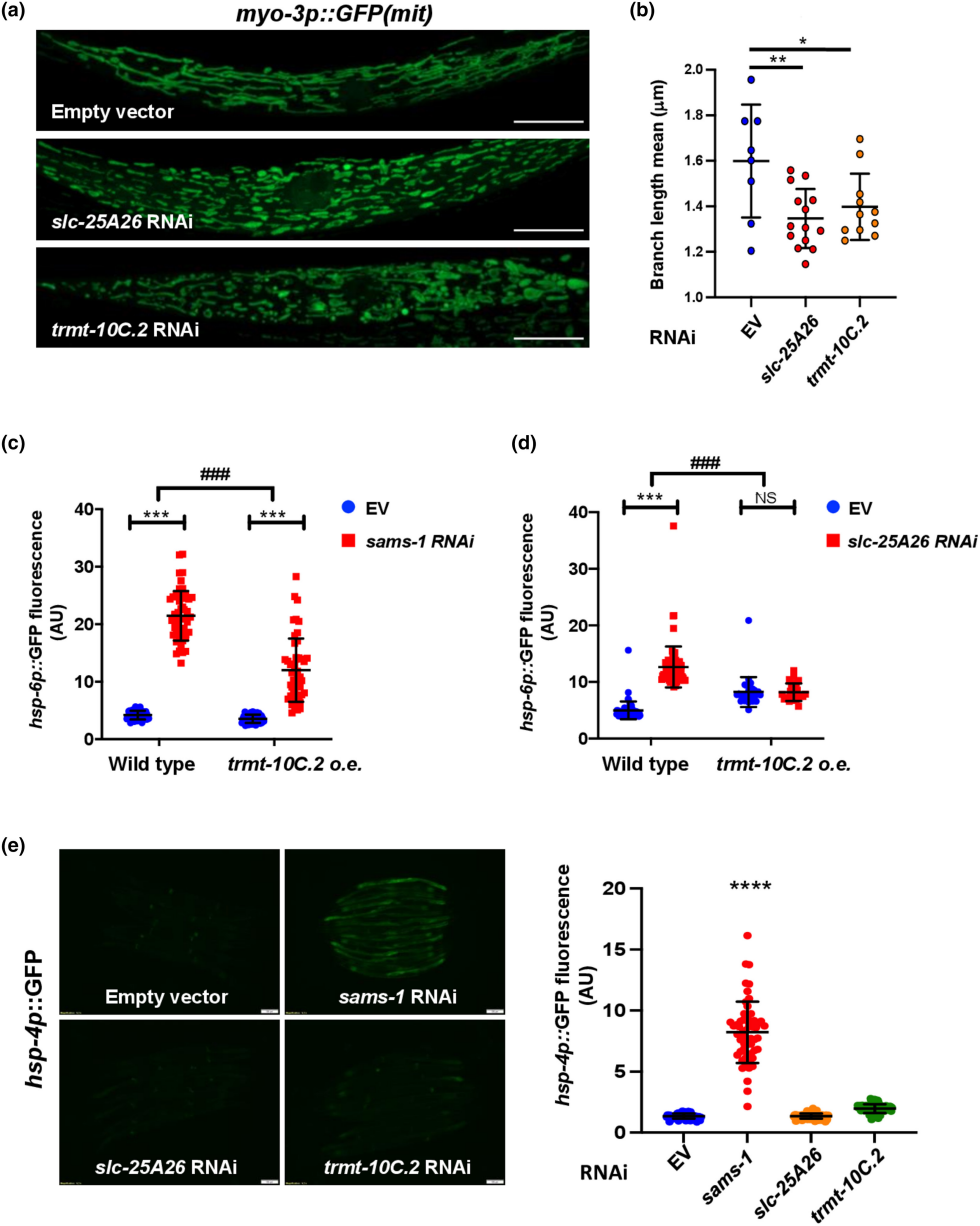
SLC‐25A26‐TRMT‐10C.2 axis serves as the downstream mediator in the regulation of mitochondrial functions by SAMS‐1. (a) Representative confocal images of the mitochondrial morphology of *myo‐3p*::mtGFP worms fed with empty vector, *slc‐25A26*, or *trmt‐10C*.*2* RNAi bacteria. Scale bar, 10 μm. (b) Average mitochondrial length (μm) in the muscle cells of *myo‐3p*::mtGFP worms treated with empty vector (EV), *slc‐25A26*, *trmt‐10C*.*2* RNAi (*p < 0.05, **p < 0.01, one‐way ANOVA). (c, d) Quantification of *hsp‐6p*::*GFP* expression in wild‐type and *trmt‐10C*.*2* overexpressing worms fed with empty vector, *sams‐1* (c), *slc‐25A26* (d) RNAi bacteria. Representative images are included in Figure S5. Data were analyzed by two‐way ANOVA with Tukey's multiple comparison test. Levels of significance are shown as ^####^ *p* < 0.0001 (interaction); ***p < 0.001 (pairwise); NS, not significant. Data are representative of at least two biological replicates with 50 worms for each treatment (*N* ≥ ; *n* ≥ 50). (e) Quantification of *hsp‐4p*::*GFP* expression in wild‐type worms fed with empty vector, *slc‐25A26*, and *trmt‐10C*.*2* RNAi bacteria. ***p < 0.001, one‐way ANOVA. Scale bar = 100 μm. Data are representative of at least two biological replicates with at least 50 worms for each treatment (*N* ≥ 2; *n* ≥ 50).

Previously, in Wei et al.'s study, they found that vitamin B12 deficiency as well as *sams‐1* RNAi both lead to SAM deficiency that consequently resulted in an increased mitochondrial fragmentation in wild‐type N2 (Wei & Ruvkun, 2020). Thus, we asked whether blocking SAM import into mitochondria may also induce mitochondria fragmentation. We first depleted *slc‐25A26* in transgenic animals carrying *myo‐3p*::mtGFP and observed the mitochondrial morphology in the body wall muscle. We found that RNAi depletion of *slc‐25A26* is sufficient to cause mitochondrial fragmentation (Figure 5a,b), suggesting that the increased mitochondrial fragmentation observed in B12 deficiency and SAM deficiency animals might, at least in part, be due to reduced mitochondrial SAM. Interestingly, RNAi knockdown of the putative tRNA methyltransferase *trmt‐10C*.*2* also caused a similar effect of increased mitochondrial fragmentation (Figure 5a,b). This result is consistent with previous findings that suppressing mitochondrial translation would increase mitochondrial fission (Houtkooper et al., 2013). To further elucidate the effects of *slc‐25A26* or *trmt‐10C*.*2* on mitochondrial fragmentation, we examined the effect of *slc‐25A26* or *trmt‐10C*.*2* RNAi knockdown in *drp‐1*(*tm1108*) mutants, as Dynamin‐related protein 1 DRP‐1 is required for mitochondria fission (Taguchi et al., 2007). Our results showed that knockdown of either *slc‐25A26* or *trmt‐10C*.*2* failed to trigger mitochondrial fragmentation in *drp‐1* mutants (Figure S4), suggesting that mitochondrial SAM deficiency may induce mitochondrial fragmentation in a *drp‐1*‐dependent manner.

Next, to examine whether the UPR^mt^ observed in SAM deficiency animals is mediated by the SLC‐25A26‐TRMT‐10C.2 axis, we assessed the *hsp‐6p*::*gfp* expression level in *trmt‐10C*.*2* overexpressing animals grown on either *sams‐1* or *slc‐25A26* RNAi bacteria. Indeed, the UPR^mt^ induced by RNAi depletion of either *sams‐1* or *slc‐25A26* was significantly decreased in transgenic animals overexpressing *trmt‐10C*.*2* (Figure 5c,d, Figure S5), suggesting that *trmt‐10C*.*2* acts downstream of SAMS‐1 and SLC‐25A26 to regulate UPR^mt^. Furthermore, apart from inducing UPR^mt^, depletion of *sams‐1* has been reported to activate endoplasmic reticulum unfolded protein response (UPR^ER^), potentially attributed to alterations in ER membrane fluidity (Hou et al., 2014). To examine whether the SLC‐25A26‐TRMT‐10C.2 axis modulates UPR^ER^ as well, we assessed UPR^ER^ using *hsp‐4p*::*GFP* reporter strain. The results indicated that RNAi depletion of *slc‐25A26* and *trmt‐10C*.*2* could not activate UPR^ER^ (Figure 5e). This observation suggests that the SLC‐25A26‐TRMT‐10C.2 axis might be highly organelle‐specific. Together, our results suggest that the reduced mitochondrial tRNA methylation and consequently the mitochondrial protein translation may be responsible for the UPR^mt^ triggered by mitochondrial SAM deficiency.

### 2.4 SAM deficiency‐induced UPR^mt^ contributes to longevity in *sams‐1* mutants

**FIGURE 6:**
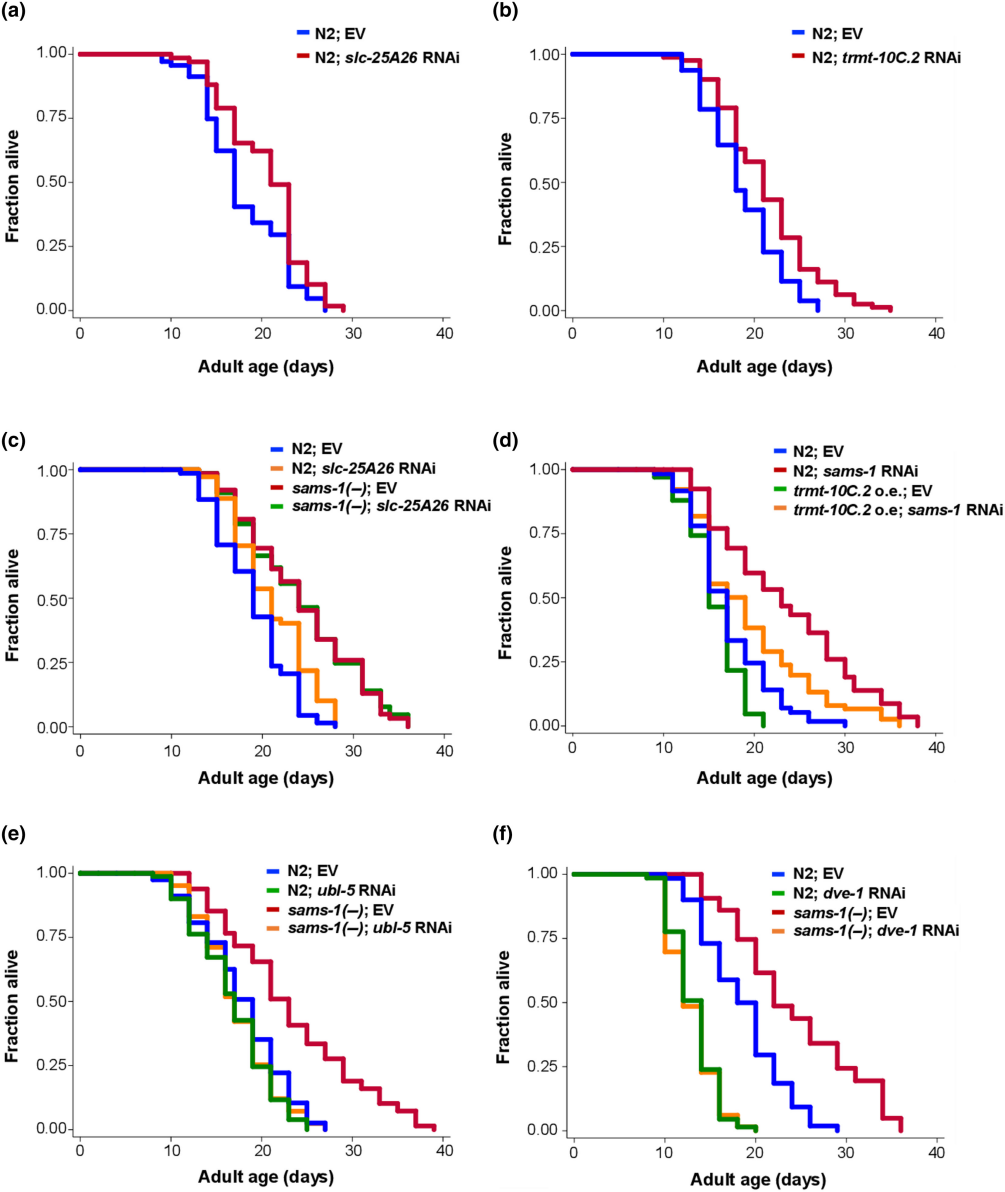
*slc‐25A26* and *trmt‐10C*.*2* are the downstream mediators of *sams‐1*‐regulated longevity. Lifespan analysis of (a) wild‐type N2 animals grown on empty vector (blue) or *slc‐25A26* (red) RNAi bacteria. (b) wild‐type N2 animals grown on empty vector (blue) or *trmt‐10C*.*2* (red) RNAi bacteria. (c) wild‐type N2 animals (blue and orange) and *sams‐1*(*ok2946*) mutants (red and green) grown on empty vector or *slc‐25A26* RNAi bacteria. (d) wild‐type N2 animals (blue and red) and *trmt‐10C*.*2* overexpressed animals (green and orange) grown on empty vector or *sams‐1* RNAi bacteria. (e) wild‐type N2 animals (blue and green) and *sams‐1*(*ok2946*) mutants (red and orange) grown on empty vector or *ubl‐5* RNAi bacteria. (f) wild‐type N2 animals (blue and green) and *sams‐1*(*ok2946*) mutants (red and orange) grown on empty vector or *dve‐1* RNAi bacteria. Additional lifespan replicates are included in Table S1.

Previous studies have shown that increased UPR^mt^ resulting from various internal or external stimuli extends lifespan in *C*. *elegans* (Bennett & Kaeberlein, 2014). We thus asked whether the SAM deficiency‐induced UPR^mt^ also affects longevity in worms. First, we performed lifespan analysis on wild‐type worms treated with either *slc‐25A26* or *trmt‐10C*.*2* RNAi. We found that the decrease in SLC‐25A26 or TRMT‐10C.2 expression resulted in a significant extension of the lifespan in wild‐type animals (Figure 6a,b), suggesting that reducing mitochondrial SAM levels indeed promotes longevity. We next examined whether SAM deficiency in mitochondria contributes to the longevity of *sams‐1* mutants. Our result showed that RNAi depletion of *slc‐25A26* cannot further extend the lifespan of *sams‐1* mutants (Figure 6c). Moreover, the lifespan extension in *sams‐1* RNAi KD animals was significantly suppressed by overexpression of *trmt‐10C*.*2* gene (Figure 6d).

We have previously reported that autophagy activity is required for the lifespan extension of *sams‐1* mutants (Lim et al., 2023). Thus, we next investigated the involvement of autophagy in lifespan extension induced by mitochondrial SAM deficiency. We performed double knockdown of *bec‐1* and *trmt‐10C*.*2* in wild‐type animals and found that the depletion of *trmt‐10C*.*2* could still extend lifespan when autophagy initiation is suppressed (Figure S6a), suggesting that *trmt‐10C*.*2* influences longevity in an autophagy‐independent manner.

As DRP‐1 is required for the mitochondrial fragmentation resulting from the depletion of *slc‐25A26* or *trmt‐10C*.*2* (Figure S4), we thus proceeded to examine the potential involvement of *drp‐*1 in the longevity phenotype associated with the SLC‐25A26‐TRMT‐10C.2 axis. RNAi depletion of *slc‐25A26* or *trmt‐10C*.*2* failed to extend lifespan in *drp‐1* null mutants (Figure S6b) suggesting that mitochondrial fragmentation is indeed required for their lifespan phenotypes. To gain further insight into the role of UPR^mt^ in SLC‐25A26‐TRMT‐10C.2‐mediated longevity, we subsequently investigated whether knocking down *slc‐25A26* or *trmt‐10C*.*2* could induce UPR^mt^ in *drp‐1* mutants. We found that depleting DRP‐1 markedly attenuated UPR^mt^ in *slc‐25A26* or *trmt‐10C*.*2* RNAi mutants (Figure S6c), supporting the idea that UPR^mt^ is critical for lifespan extension by SLC‐25A26 and TRMT‐10C.2. Finally, we knocked down *ubl‐5* or *dve‐1*, two essential transcription factors in UPR^mt^, to further examine the role of UPR^mt^ in SAM deficiency‐mediated longevity. Our results indicated that both *ubl‐5* and *dve‐1* are required for lifespan extension in *sams‐1* mutants (Figure 6e,f), reaffirming the significance of UPR^mt^ in the context of longevity influenced by SAM deficiency. As knockdown of *ubl‐5* is known to cause mitochondrial fragmentation but inhibits UPR^mt^ (Benedetti et al., 2006), our findings together suggest that mitochondrial SAM deficiency (e.g., *sams‐1*, *slc‐25A26* RNAi) may trigger mitochondrial fragmentation first and consequently the UPR^mt^, which are both required for lifespan extension.

## 3 DISCUSSION

It is a commonly accepted concept that a well‐maintained SAM homeostasis is critical for any organelle that carries out SAM‐dependent methylation reactions. This of course includes mitochondria, a key organelle for numerous cellular functions. Severe genetic defects in human *SLC25A26*, the mitochondrial SAM carrier, have been reported to cause neonatal mortality (Kishita et al., 2015). Our findings in *C*. *elegans* have also demonstrated that inhibiting worm *slc‐25A26*, which significantly reduced mitochondrial SAM levels, markedly affects mitochondrial morphology and respiration. Together, these findings strongly suggest that SAM homeostasis in mitochondria is crucial for normal cellular functions.

While the functions of many mitochondrial methyltransferases (MTs) have been well‐studied, the underlying molecular mechanisms controlling mitochondrial SAM homeostasis remain largely unexplored. In particular, how dietary changes impact mitochondrial SAM homeostasis remains unclear. It has been previously shown that SAMS‐1 expression is significantly reduced in response to starvation (Hansen et al., 2005; Harvald et al., 2017). Since SAMS‐1 is the primary enzyme responsible for the biosynthesis of SAM in *C*. *elegans* (Walker et al., 2011), deficiency in SAMS‐1 would likely impact SAM homeostasis both in the cytoplasm and in the mitochondria. Indeed, in this study, we found that SAM levels in the mitochondria are significantly lowered when SAMS‐1 is depleted. Notably, methionine restriction in mice reduces SAM levels in the serum and the liver (Roy et al., 2020). Therefore, the cellular SAM levels, particularly the mitochondrial SAM level, may serve as a sensor for dietary changes, so that cells can make necessary adjustments to cope with these changes (e.g., reduced protein translations in both mitochondria).

UPR^mt^ is a cellular response required to maintain the normal functions of the mitochondrial network. It is well‐known that perturbations in mitochondrial proteostasis and impairments of mitochondrial functions, such as oxidative phosphorylation, could activate the UPR^mt^ (Melber & Haynes, 2018). It has been previously demonstrated that the inactivation of *sams‐1*, which leads to lower SAM levels, induced a robust mitochondrial UPR^mt^ in worms (Hou et al., 2014). Similarly, it has been shown that vitamin B12 deficiency, which results in a low methionine level, activates UPR^mt^ in *C*. *elegans* (Wei & Ruvkun, 2020). Together with our findings in this study, the increased UPR^mt^ observed in methionine‐ or SAM‐deficient animals may likely be a result of the loss of SAM homeostasis and altered SAM‐dependent methylations in the mitochondria. Intriguingly, while there are more than a dozen methyltransferases predicted to be localized in the mitochondria to carry out numerous methylation reactions in *C*. *elegans*, depleting the putative mt‐tRNA methyltransferases *trmt‐10C*.*2* alone is sufficient to trigger a strong UPR^mt^. As disruption of mitochondrial tRNA methylation likely leads to an impairment of mitochondrial tRNA maturation, which will consequently result in a reduced mitochondrial translation, our results suggest that mitochondrial translation seems to be much more sensitive to the shortage of SAM than other processes involved in methylation reactions in the mitochondria. It is recognized that the assembly of respiratory chain complexes relies on the intricate coordination between the biogenesis of mitochondria‐encoded and nuclear‐encoded components (Priesnitz & Becker, 2018). Furthermore, multiple studies in *C*. *elegans* have shown that disruption of genes associated with mitochondrial translation robustly induces the UPR^mt^, possibly due to disruption of the stoichiometry of respiratory complexes (Bennett et al., 2014; Rolland et al., 2019; Suhm & Ott, 2017). Our research, along with other studies, collectively emphasizes the significance of maintaining mitochondrial protein synthesis for the proper function of mitochondria.

It has been reported that dietary methionine restriction affects mitochondrial functions and increases lifespan in various organisms (McIsaac et al., 2016; Naudi et al., 2007; Sanchez‐Roman & Barja, 2013). Moreover, lowering SAM levels via genetic interventions extends lifespan in both worms and fruit flies (Hansen et al., 2005; Obata & Miura, 2015). As a universal methyl donor, SAM is required in many metabolic and epigenetic processes. We have shown in our recent studies that limiting the availability of nuclear SAM activates autophagy and increases lifespan via inhibiting SET‐2, an H3K4 methyltransferase, in *C*. *elegans* (Lim et al., 2023). The alteration in H3K4 tri‐methylation status enhances the affinity of HLH‐30 and PHA‐4 for the promoter of autophagy‐related genes, leading to an extension in lifespan. Here, we further demonstrated that deficiency in mitochondrial SAM level might influence lifespan by modulating UPR^mt^. Thus, the pro‐longevity effects observed in dietary methionine‐restricted or *sams‐1* mutant animals may result from a synergy of the molecular consequences of SAM deficiency in both nucleus and mitochondria. It is worth noting that *hsp‐6* and *hsp‐60*, two crucial chaperone proteins involved in UPR^mt^, possess binding sites for HLH‐30 and PHA‐4 within their promoter regions (Oki et al., 2018; Zhong et al., 2010). This observation hints at a potential coordinated UPR^mt^ response in both nucleus and mitochondria during SAM deficiency. Further investigation on whether chromatin remodeling induced by nuclear SAM deficiency is necessary for UPR^mt^ in the presence of mitochondrial SAM deficiency would help clarify this matter.

Mutations in MATA1a or SLC25A26 cause various defects in the mammalian system. Similarly, strong deletion alleles of *sams‐1*, while extending lifespan, exhibit many negative pleiotropic phenotypes, such as developmental abnormalities, reproductive dysfunction, and impaired lipid metabolism. In this study, lifespan extensions were observed when these genes were knocked down by RNAi only during adulthood to avoid potential adverse developmental phenotypes. Therefore, we believe that, as also observed in many other longevity regulatory pathways, there are both dosage and temporal requirements for the manipulation of mitochondrial SAM levels to be a potential target for anti‐aging intervention without severe trade‐offs.

## 4 EXPERIMENTAL PROCEDURES

### 4.1 Strains

N2: wild type.

CLP334: *zcls14*[*myo‐3p*::*GFP*(*mit*)]; *drp‐1*(*tm1108*).

SJ4005: *zcIs4* [*hsp‐4*::*GFP*] (Bennett et al., 2014).

SJ4100: *zcIs13* [*hsp‐6p*::*GFP*].

SJ4103: *zcIs14* [*myo‐3p*::*GFP*(*mit*)].

EQ153: *sams‐1*(*ok2946*).

EQ1183: *iqEx246*[*trmt‐10C*.*2p*::*trmt‐10C*.*2 + myo‐3p*::*RFP*].

EQ1195: *iqEx248*[*trmt‐10C*.*2p*:: *trmt‐10C*.*2*::*GFP + myo‐2p*::*tdTomato*].

EQ1642: *zcIs*[*hsp‐6p*::*GFP*]; *iqEx327*[*trmt10C*.*2p*::*trmt‐10C*.*2+ myo‐2p*::*tdTomato*].

EQ1734: *iqEx347*[*ges‐1p*::*slc‐25A26*::*GFP + myo‐2p*::*tdTomato*].

EQ1792: *iqEx347*[*ges‐1p*::*slc25A26*::*GFP + myo‐2p*::*tdTomato*]; *iqIs276* [*ges‐1p*::*tomm‐20*::*mCherry + rol‐6p*::*rol‐6*].

EQ1941: *igEx398*[*slc25A26p*::*GFP + rol‐6p*::*rol‐6*].

EQ2028: *drp‐1*(*tm1108*) (back‐crossed to Hsu lab N2).

SJ4005, SJ4100, SJ4103, and wild‐type *C*. *elegans* (N2) strains were obtained from the Caenorhabditis Genetic Center. CLP334 worms were a generous gift from Dr. Chun‐Liang Pan. All strains were maintained under standard culturing conditions.

### 4.2 RNA‐interference (RNAi) clones

The identity of all RNAi clones was verified by sequencing the inserts using M13‐forward primer. All clones were from Julie Ahringer's RNAi library. HT115 bacteria transformed with RNAi vectors expressing dsRNA of the genes of interest were grown at 37°C in LB with 10 μg/mL tetracycline and 50 μg/mL carbenicillin, then seeded onto NG‐carbenicillin plates and supplemented with 1 mM IPTG.

### 4.3 Brood size analysis

Single L4 larvae worms were isolated on each plate seeded with L4440 (empty vector), *slc‐25A26* RNAi, or *trmt‐10C*.*2* RNAi bacteria. Worms were transferred to fresh bacterial plates daily until the end of ovulation. The number of eggs was recorded every day. The brood size was determined by summing up of total eggs laid by individual worms.

### 4.4 Developmental rate

Synchronized worms were generated by allowing 20 Day 1 adult worms to lay eggs for 4 h. Worms were kept at 20°C. Images were taken every 24 h until the worms reached adulthood. Body length was measured by image J.

### 4.5 Microscopy analysis

To determine UPR^mt^ or UPR^ER^ activation, strains carrying *hsp‐6p*::*GFP* or *hsp‐4p*::*GFP* were utilized. Synchronized worms were visualized at of day 1 of adulthood. For the fluorescence imaging, worms were anesthetized with M9 buffer containing 20 mM sodium azide, and an Olympus BX63 microscope was used. Worm GFP intensity was then quantified with ImageJ software. In SAM, methionine, and homocysteine supplementation experiments, P_0_ worms were grown on empty vector or RNAi bacteria from hatching. F_1_ eggs were then transferred on plates seeded with UV‐killed bacteria and supplemented with the vehicle, 2 mM SAM (Sigma‐Aldrich, A2408), 2 mM L‐methionine (Sigma‐Aldrich, M5308), and 2 mM homocysteine (Sigma‐Aldrich, 69453). Day 1 adult F_1_ worms were then scored for GFP intensity.

For monitoring the subcellular localization of SLC‐25A26::GFP or TRMT‐10C.2::GFP, worms were mounted on 2% agarose pads with 50 mM tetramisole (Sigma, T1512). Images were acquired using Zeiss Elyra 7 microscope and Zeiss LSM 900 laser scanning confocal microscope, respectively.

For Mitotracker Red staining, L4 worms carrying TRMT‐10C.2::GFP were cultured on UV‐killed bacteria with 2 μM Mitotracker (Cell Signaling Technology, 8778) for 24 h. Worms were subsequently transferred to stain‐free plates for 30 min to eliminate the excess stain in the gut lumen before proceeding with image analysis.

### 4.6 Mitochondria isolation

Mitochondria isolation was described as previous. In brief, up to 20,000 adult day 1 worms were grown on 10 X 100 mm nematode growth medium (NGM) plates seeded with *Escherichia coli* OP50 bacteria. Worms were harvested by ice‐cold M9 supplemented with Triton X‐100, followed by 15 mL M9 buffer to wash off bacteria in Falcon tube. The worms were then washed once with 15 mL ddH2O, and resuspended in 1 mL ice‐cold mitochondria isolation buffer [MIB; 50 mM KCl, 110 mM mannitol, 70 mM sucrose, 0.1 mM EDTA (pH 8.0), 5 mM Tris–HCl (pH 7.4)], supplemented with protease inhibitors and phosphatase inhibitors (Roche). Worms were transferred into a 7 mL Dounce homogenizer and homogenized with 30 gentle strokes of the piston on ice. The samples were then centrifuged (200 × g for 5 min followed by 1000 × g for 10 min) to discard debris. After passing through the syringe filter (Sartorius, 17593‐K) to remove potential nucleus contamination, the isolated mitochondria were pelleted by high‐speed centrifugation (12,000 × g for 10 min) and were ready for further examination.

### 4.7 SAM level analysis

S‐adenosylmethionine (SAM) levels were measured by S‐Adenosylmethionine ELISA kit (Cell Biolabs, MET‐5151c). In brief, isolated mitochondria were lysed by sonication and then subjected to 96‐well plate coated with SAM conjugate. The samples were then agitated with anti‐SAM assay diluent (1:1000) at room temperature for 1 h and washed three times with washing buffer. The samples were then probed with HRP‐conjugated secondary antibodies for 1 h, followed by washing three times with washing buffer. After adding the substrate solution, samples were incubated in the dark at room temperature for 20 min. Stop solution was then added to the samples to quench signal saturation. The absorbance value was measured at 450 nm with 620 nm reference wavelength using TECAN Infinite M200 pro spectrophotometer.

### 4.8 Mitochondrial oxygen consumption assays

Mitochondrial Oxygen Consumption Rate (OCR) was measured using Seahorse XFe24 analyzer (Agilent Technologies) based on the previously described protocol with slight modification. In brief, synchronized worms were grown on NGM plates at 20°C. Worms were then picked to the 24‐well plates containing 500 μL M9 buffer in a density of 30 worms per well. Basal OCR was measured six times, followed by the addition of 10 μM of FCCP (Sigma‐Aldrich, C2920), and measurements of maximal respiratory capacity were performed another 6 times with 6‐min intervals. Nonmitochondrial respiration rates were then measured after supplementation of 50 mM of sodium azide (Sigma‐Aldrich, S2002).

### 4.9 Western blotting analysis

Western blot of CTC‐1 and NUO‐2 protein in *C*. *elegans* was performed by running the worm lysates on SDS‐PAGE gels and followed by immunoblotting using the following antibodies: anti‐CTC‐1 (Abcam, 14705), anti‐NUO‐2 (Abcam, 14711), and anti‐actin (MP Biomedicals, 691001). Blots were developed using SuperSignal West Pico Plus chemiluminescent substrate (Thermo Fisher) and visualized using ChemiDoc MP imaging system (Bio‐Rad). Relative protein levels were determined using ImageJ densitometry of immunoblots.

### 4.10 Mitochondrial puromycin incorporation assay

Ten thousand day 1 worms were collected and then soaked in M9 buffer with 1 mg/mL puromycin for 15 min at room temperature with gentle agitation. Worms were then washed three times with M9 to remove additional puromycin. Samples were proceeded with mitochondria isolation. Protein amounts of the whole worm lysate and mitochondrial fraction were determined by DC protein assay kit (Bio‐Rad). Anti‐puromycin antibody (Merck, MABE343) was used to detect incorporated puromycin. Anti‐NUO‐2 and anti‐actin were used as the loading control of the mitochondrial lysate and whole worm lysate, respectively.

### 4.11 Lifespan analysis

Lifespan analysis was conducted at 20°C as described previously (Hsu et al., 2003) unless otherwise stated. Strains were grown at 20°C for at least two generations before the experiments. 60–90 animals were tested in each experiment. RNAi treatments were carried out by adding synchronized L4 worms to plates seeded with the RNAi bacteria of interest. Worms were moved to fresh RNAi plates every 2 days until reproduction ceased. Worms were then moved to new plates every 5–7 days for the rest of the lifespan analysis. The viability of the worms was scored every 2 days. In all experiments, the pre‐fertile period of adulthood was used as *t* = 0 for life span analysis. Stata 12 (StataCorp) software was used for statistical analysis to determine the means and percentiles. In all cases, *p* values were calculated using the log‐rank (Mantel‐Cox) method.

## AUTHOR CONTRIBUTIONS

T‐T. C. conceived the project and designed the experiments. T‐Y. C., F‐Y. W., and P‐J. L. performed the experiments and analyzed the data. T‐T. C. and A‐L. H. interpreted the data. T‐T. C. and A‐L. H. wrote and edited the manuscript.

## CONFLICT OF INTEREST STATEMENT

The authors declare no competing financial interests.

## Supporting information


Figure S1.

Figure S2.

Figure S3.

Figure S4.

Figure S5.

Figure S6.

Table S1.

Data S1.

## Data Availability

The data that support the findings of this study are available from the corresponding author upon reasonable request.

## References

[acel14103-bib-0001] Agrimi, G. , Di Noia, M. A. , Marobbio, C. M. , Fiermonte, G. , Lasorsa, F. M. , & Palmieri, F. (2004). Identification of the human mitochondrial S‐adenosylmethionine transporter: Bacterial expression, reconstitution, functional characterization and tissue distribution. The Biochemical Journal, 379(Pt 1), 183–190. 10.1042/BJ20031664 14674884 PMC1224042

[acel14103-bib-0002] Benedetti, C. , Haynes, C. M. , Yang, Y. , Harding, H. P. , & Ron, D. (2006). Ubiquitin‐like protein 5 positively regulates chaperone gene expression in the mitochondrial unfolded protein response. Genetics, 174(1), 229–239. 10.1534/genetics.106.061580 16816413 PMC1569816

[acel14103-bib-0003] Bennett, C. F. , & Kaeberlein, M. (2014). The mitochondrial unfolded protein response and increased longevity: Cause, consequence, or correlation? Experimental Gerontology, 56, 142–146. 10.1016/j.exger.2014.02.002 24518875 PMC4048780

[acel14103-bib-0004] Bennett, C. F. , Vander Wende, H. , Simko, M. , Klum, S. , Barfield, S. , Choi, H. , Pineda, V. V. , & Kaeberlein, M. (2014). Activation of the mitochondrial unfolded protein response does not predict longevity in *Caenorhabditis elegans* . Nature Communications, 5, 3483. 10.1038/ncomms4483 PMC3984390 24662282

[acel14103-bib-0005] Chen, C. C. , Lim, C. Y. , Lee, P. J. , Hsu, A. L. , & Ching, T. T. (2020). S‐adenosyl methionine synthetase SAMS‐5 mediates dietary restriction‐induced longevity in *Caenorhabditis elegans* . PLoS One, 15(11), 0241455. 10.1371/journal.pone.0241455 PMC7657561 33175851

[acel14103-bib-0006] Cheng, G. P. , Guo, S. M. , Yin, Y. , Li, Y. Y. , He, X. , & Zhou, L. Q. (2022). Aberrant expression of mitochondrial SAM transporter SLC25A26 impairs oocyte maturation and early development in mice. Oxidative Medicine and Cellular Longevity, 2022, 1681623. 10.1155/2022/1681623 35464759 PMC9020962

[acel14103-bib-0007] Godbole, A. A. , Gopalan, S. , Nguyen, T. K. , Munden, A. L. , Lui, D. S. , Fanelli, M. J. , Vo, P. , Lewis, C. A. , Spinelli, J. B. , Fazzio, T. G. , & Walker, A. K. (2023). S‐adenosylmethionine synthases specify distinct H3K4me3 populations and gene expression patterns during heat stress. eLife, 12, 79511. 10.7554/eLife.79511 PMC9984191 36756948

[acel14103-bib-0008] Hansen, M. , Hsu, A. L. , Dillin, A. , & Kenyon, C. (2005). New genes tied to endocrine, metabolic, and dietary regulation of lifespan from a *Caenorhabditis elegans* genomic RNAi screen. PLoS Genetics, 1(1), 119–128. 10.1371/journal.pgen.0010017 16103914 PMC1183531

[acel14103-bib-0009] Harvald, E. B. , Sprenger, R. R. , Dall, K. B. , Ejsing, C. S. , Nielsen, R. , Mandrup, S. , Murillo, A. B. , Larance, M. , Gartner, A. , Lamond, A. I. , & Faergeman, N. J. (2017). Multi‐omics analyses of starvation responses reveal a central role for lipoprotein metabolism in acute starvation survival in *C*. *Elegans* . Cell Systems, 5(1), 38–52. 10.1016/j.cels.2017.06.004 28734827

[acel14103-bib-0010] Hou, N. S. , Gutschmidt, A. , Choi, D. Y. , Pather, K. , Shi, X. , Watts, J. L. , Hoppe, T. , & Taubert, S. (2014). Activation of the endoplasmic reticulum unfolded protein response by lipid disequilibrium without disturbed proteostasis in vivo. Proceedings of the National Academy of Sciences of the United States of America, 111(22), E2271–E2280. 10.1073/pnas.1318262111 24843123 PMC4050548

[acel14103-bib-0011] Houtkooper, R. H. , Mouchiroud, L. , Ryu, D. , Moullan, N. , Katsyuba, E. , Knott, G. , Williams, R. W. , & Auwerx, J. (2013). Mitonuclear protein imbalance as a conserved longevity mechanism. Nature, 497(7450), 451–457. 10.1038/nature12188 23698443 PMC3663447

[acel14103-bib-1002] Hsu, A. L. , Murphy, C. , & Kenyon, C. (2003). Regulation of aging and age‐related disease by DAF‐16 and heat‐shock factor. Science, 300(5622), 1142–1145. 12750521 10.1126/science.1083701

[acel14103-bib-0012] Kishita, Y. , Pajak, A. , Bolar, N. A. , Marobbio, C. M. , Maffezzini, C. , Miniero, D. V. , Monné, M. , Kohda, M. , Stranneheim, H. , Murayama, K. , Naess, K. , Lesko, N. , Bruhn, H. , Mourier, A. , Wibom, R. , Nennesmo, I. , Jespers, A. , Govaert, P. , Ohtake, A. , … Wedell, A. (2015). Intra‐mitochondrial methylation deficiency due to mutations in SLC25A26. American Journal of Human Genetics, 97(5), 761–768. 10.1016/j.ajhg.2015.09.013 26522469 PMC4667130

[acel14103-bib-0013] Kotb, M. , Mudd, S. H. , Mato, J. M. , Geller, A. M. , Kredich, N. M. , Chou, J. Y. , & Cantoni, G. L. (1997). Consensus nomenclature for the mammalian methionine adenosyltransferase genes and gene products. Trends in Genetics, 13(2), 51–52. 9055605 10.1016/s0168-9525(97)01013-5

[acel14103-bib-0014] Lim, C. Y. , Lin, H. T. , Kumsta, C. , Lu, T. C. , Wang, F. Y. , Kang, Y. H. , Hansen, M. , Ching, T. T. , & Hsu, A. L. (2023). SAMS‐1 coordinates HLH‐30/TFEB and PHA‐4/FOXA activities through histone methylation to mediate dietary restriction‐induced autophagy and longevity. Autophagy, 19(1), 224–240. 10.1080/15548627.2022.2068267 35503435 PMC9809948

[acel14103-bib-0015] McIsaac, R. S. , Lewis, K. N. , Gibney, P. A. , & Buffenstein, R. (2016). From yeast to human: Exploring the comparative biology of methionine restriction in extending eukaryotic life span. Annals of the New York Academy of Sciences, 1363, 155–170. 10.1111/nyas.13032 26995762

[acel14103-bib-0016] Melber, A. , & Haynes, C. M. (2018). UPR(mt) regulation and output: A stress response mediated by mitochondrial‐nuclear communication. Cell Research, 28(3), 281–295. 10.1038/cr.2018.16 29424373 PMC5835775

[acel14103-bib-0017] Metodiev, M. D. , Thompson, K. , Alston, C. L. , Morris, A. A. , He, L. , Assouline, Z. , Rio, M. , Bahi‐Buisson, N. , Pyle, A. , Griffin, H. , Siira, S. , Filipovska, A. , Munnich, A. , Chinnery, P. F. , McFarland, R. , Rötig, A. , Taylor, R. W. , & Taylor, R. W. (2016). Recessive mutations in TRMT10C cause defects in mitochondrial RNA processing and multiple respiratory chain deficiencies. American Journal of Human Genetics, 99(1), 246. 10.1016/j.ajhg.2016.06.013 PMC5005466 27392079

[acel14103-bib-0018] Motorin, Y. , & Helm, M. (2011). RNA nucleotide methylation. Wiley Interdisciplinary Reviews: RNA, 2(5), 611–631. 10.1002/wrna.79 21823225

[acel14103-bib-0019] Naudi, A. , Caro, P. , Jove, M. , Gomez, J. , Boada, J. , Ayala, V. , Portero‐Otín, M. , Barja, G. , & Pamplona, R. (2007). Methionine restriction decreases endogenous oxidative molecular damage and increases mitochondrial biogenesis and uncoupling protein 4 in rat brain. Rejuvenation Research, 10(4), 473–484. 10.1089/rej.2007.0538 17716000

[acel14103-bib-0020] Obata, F. , & Miura, M. (2015). Enhancing S‐adenosyl‐methionine catabolism extends Drosophila lifespan. Nature Communications, 6, 8332. 10.1038/ncomms9332 PMC4595730 26383889

[acel14103-bib-0021] Oerum, S. , Roovers, M. , Rambo, R. P. , Kopec, J. , Bailey, H. J. , Fitzpatrick, F. , Newman, J. A. , Newman, W. G. , Amberger, A. , Zschocke, J. , Droogmans, L. , Oppermann, U. , & Yue, W. W. (2018). Structural insight into the human mitochondrial tRNA purine N1‐methyltransferase and ribonuclease P complexes. The Journal of Biological Chemistry, 293(33), 12862–12876. 10.1074/jbc.RA117.001286 29880640 PMC6102140

[acel14103-bib-0022] Oki, S. , Ohta, T. , Shioi, G. , Hatanaka, H. , Ogasawara, O. , Okuda, Y. , Kawaji, H. , Nakaki, R. , Sese, J. , & Meno, C. (2018). ChIP‐Atlas: A data‐mining suite powered by full integration of public ChIP‐seq data. EMBO Reports, 19(12), 46255. 10.15252/embr.201846255 PMC6280645 30413482

[acel14103-bib-0023] Petrossian, T. C. , & Clarke, S. G. (2011). Uncovering the human methyltransferasome. Molecular & Cellular Proteomics, 10(1), M110 000976. 10.1074/mcp.M110.000976 PMC3013446 20930037

[acel14103-bib-0024] Priesnitz, C. , & Becker, T. (2018). Pathways to balance mitochondrial translation and protein import. Genes & Development, 32(19–20), 1285–1296. 10.1101/gad.316547.118 30275044 PMC6169841

[acel14103-bib-0025] Rhein, V. F. , Carroll, J. , Ding, S. , Fearnley, I. M. , & Walker, J. E. (2013). NDUFAF7 methylates arginine 85 in the NDUFS2 subunit of human complex I. The Journal of Biological Chemistry, 288(46), 33016–33026. 10.1074/jbc.M113.518803 24089531 PMC3829151

[acel14103-bib-0026] Rolland, S. G. , Schneid, S. , Schwarz, M. , Rackles, E. , Fischer, C. , Haeussler, S. , Regmi, S. G. , Yeroslaviz, A. , Habermann, B. , Mokranjac, D. , Lambie, E. , & Conradt, B. (2019). Compromised mitochondrial protein import acts as a signal for UPR(mt). Cell Reports, 28(7), 1659–1669. 10.1016/j.celrep.2019.07.049 31412237

[acel14103-bib-0027] Roy, D. G. , Chen, J. , Mamane, V. , Ma, E. H. , Muhire, B. M. , Sheldon, R. D. , Shorstova, T. , Koning, R. , Johnson, R. M. , Esaulova, E. , Williams, K. S. , Hayes, S. , Steadman, M. , Samborska, B. , Swain, A. , Daigneault, A. , Chubukov, V. , Roddy, T. P. , Foulkes, W. , … Jones, R. G. (2020). Methionine metabolism shapes T helper cell responses through regulation of epigenetic reprogramming. Cell Metabolism, 31(2), 250–266. 10.1016/j.cmet.2020.01.006 32023446

[acel14103-bib-0028] Sanchez, M. I. , Mercer, T. R. , Davies, S. M. , Shearwood, A. M. , Nygard, K. K. , Richman, T. R. , Mattick, J. S. , Rackham, O. , & Filipovska, A. (2011). RNA processing in human mitochondria. Cell Cycle, 10(17), 2904–2916. 10.4161/cc.10.17.17060 21857155

[acel14103-bib-0029] Sanchez‐Roman, I. , & Barja, G. (2013). Regulation of longevity and oxidative stress by nutritional interventions: Role of methionine restriction. Experimental Gerontology, 48(10), 1030–1042. 10.1016/j.exger.2013.02.021 23454735

[acel14103-bib-0030] Schober, F. A. , Tang, J. X. , Sergeant, K. , Moedas, M. F. , Zierz, C. M. , Moore, D. , Smith, C. , Lewis, D. , Guha, N. , Hopton, S. , Falkous, G. , Lam, A. , Pyle, A. , Poulton, J. , Gorman, G. S. , Taylor, R. W. , & Wredenberg, A. (2022). Pathogenic SLC25A26 variants impair SAH transport activity causing mitochondrial disease. Human Molecular Genetics, 31(12), 2049–2062. 10.1093/hmg/ddac002 35024855 PMC9239748

[acel14103-bib-0031] Shpilka, T. , & Haynes, C. M. (2018). The mitochondrial UPR: Mechanisms, physiological functions and implications in ageing. Nature Reviews. Molecular Cell Biology, 19(2), 109–120. 10.1038/nrm.2017.110 29165426

[acel14103-bib-0032] Suhm, T. , & Ott, M. (2017). Mitochondrial translation and cellular stress response. Cell and Tissue Research, 367(1), 21–31. 10.1007/s00441-016-2460-4 27425851

[acel14103-bib-0033] Taguchi, N. , Ishihara, N. , Jofuku, A. , Oka, T. , & Mihara, K. (2007). Mitotic phosphorylation of dynamin‐related GTPase Drp1 participates in mitochondrial fission. The Journal of Biological Chemistry, 282(15), 11521–11529. 10.1074/jbc.M607279200 17301055

[acel14103-bib-0034] Walker, A. K. , Jacobs, R. L. , Watts, J. L. , Rottiers, V. , Jiang, K. , Finnegan, D. M. , Shioda, T. , Hansen, M. , Yang, F. , Niebergall, L. J. , Vance, D. E. , Tzoneva, M. , Hart, A. C. , & Naar, A. M. (2011). A conserved SREBP‐1/phosphatidylcholine feedback circuit regulates lipogenesis in metazoans. Cell, 147(4), 840–852. 10.1016/j.cell.2011.09.045 22035958 PMC3384509

[acel14103-bib-0035] Wei, W. , & Ruvkun, G. (2020). Lysosomal activity regulates *Caenorhabditis elegans* mitochondrial dynamics through vitamin B12 metabolism. Proceedings of the National Academy of Sciences of the United States of America, 117(33), 19970–19981. 10.1073/pnas.2008021117 32737159 PMC7443905

[acel14103-bib-0036] Zhong, M. , Niu, W. , Lu, Z. J. , Sarov, M. , Murray, J. I. , Janette, J. , Raha, D. , Sheaffer, K. L. , Lam, H. Y. , Preston, E. , Slightham, C. , Hillier, L. W. , Brock, T. , Agarwal, A. , Auerbach, R. , Hyman, A. A. , Gerstein, M. , Mango, S. E. , Kim, S. K. , … Snyder, M. (2010). Genome‐wide identification of binding sites defines distinct functions for *Caenorhabditis elegans* PHA‐4/FOXA in development and environmental response. PLoS Genetics, 6(2), 1000848. 10.1371/journal.pgen.1000848 PMC2824807 20174564

