# Supplementary figures and images for: Mitochondrial S‐adenosylmethionine deficiency induces mitochondrial unfolded protein response and extends lifespan in Caenorhabditis elegans
Source: Aging Cell. 2024 Feb 15;23(4):e14103. doi: 10.1111/acel.14103 (PMC11019128; doi:10.1111/acel.14103)

# Figure S1

**A**

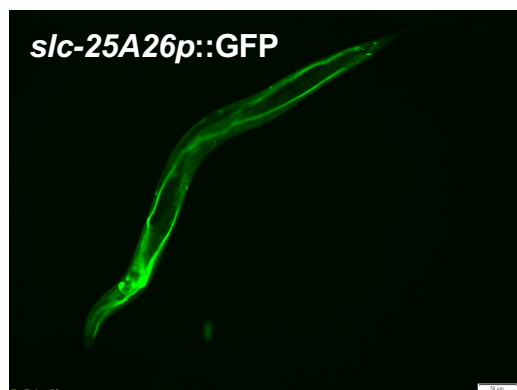

**B**

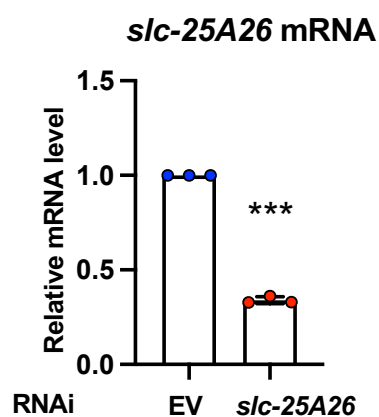

**C**

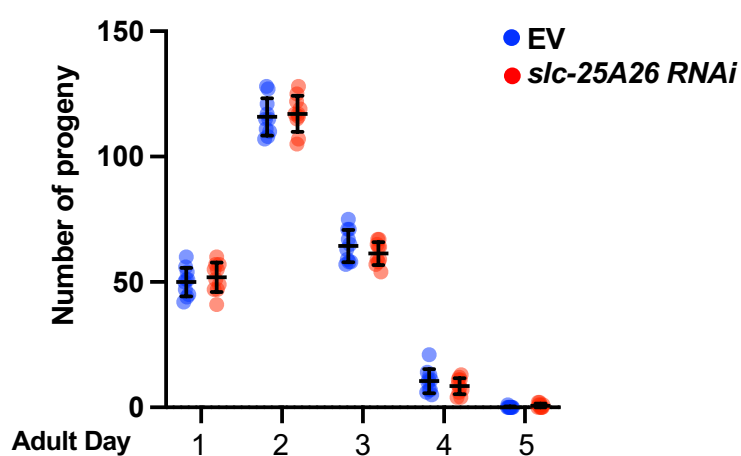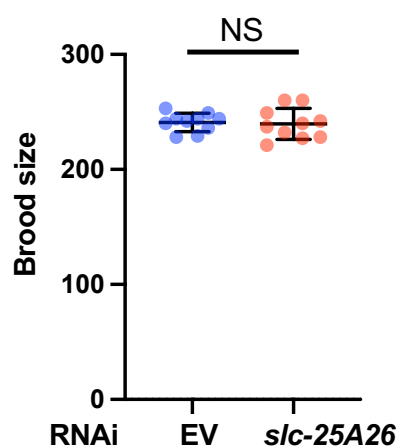

**D**

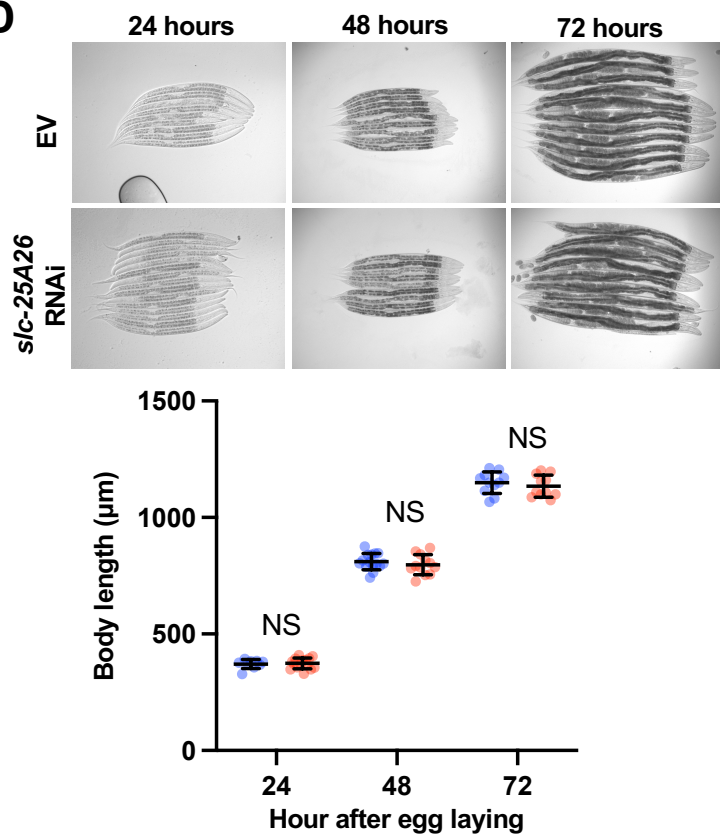

**E**

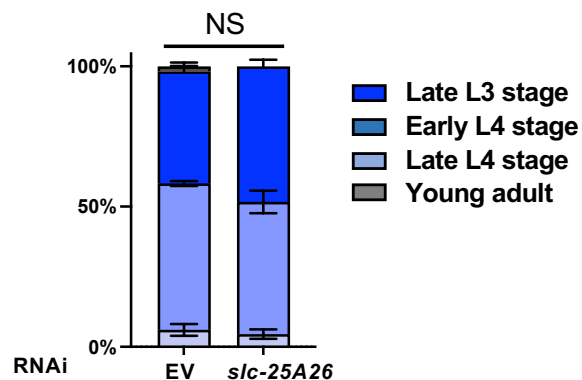

**F**

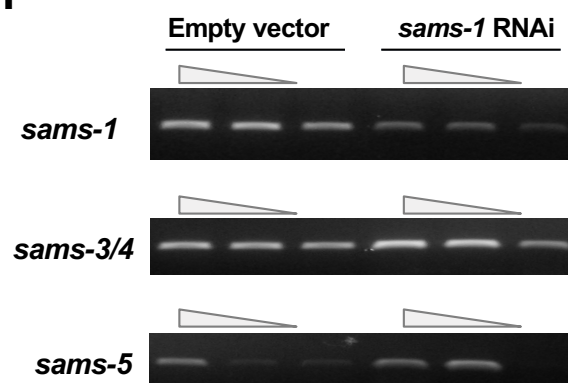

Supplement: Supplementary file 1 — Figure S1. [file ACEL-23-e14103-s008.pdf]

## Figure S2

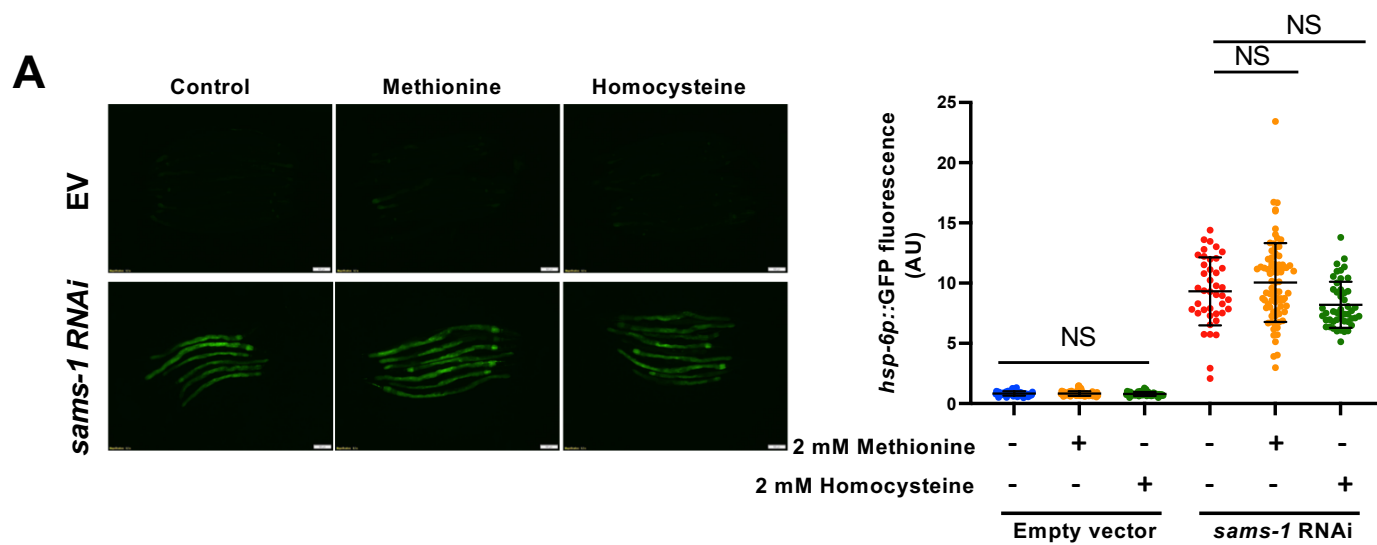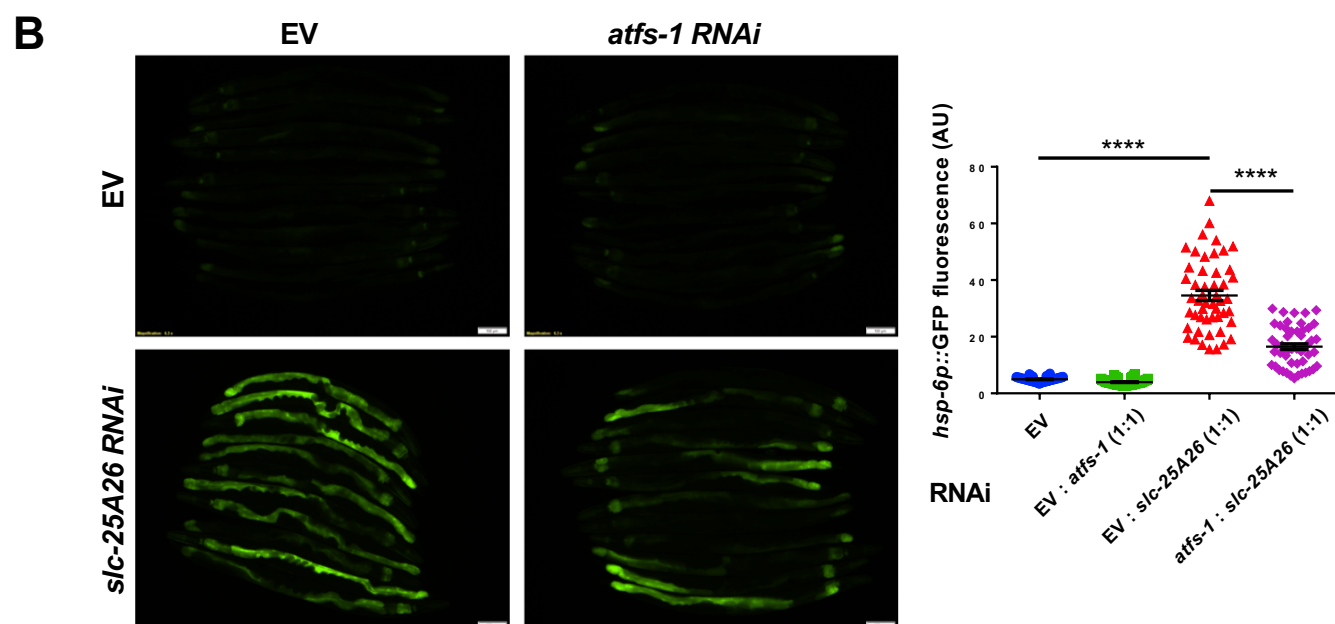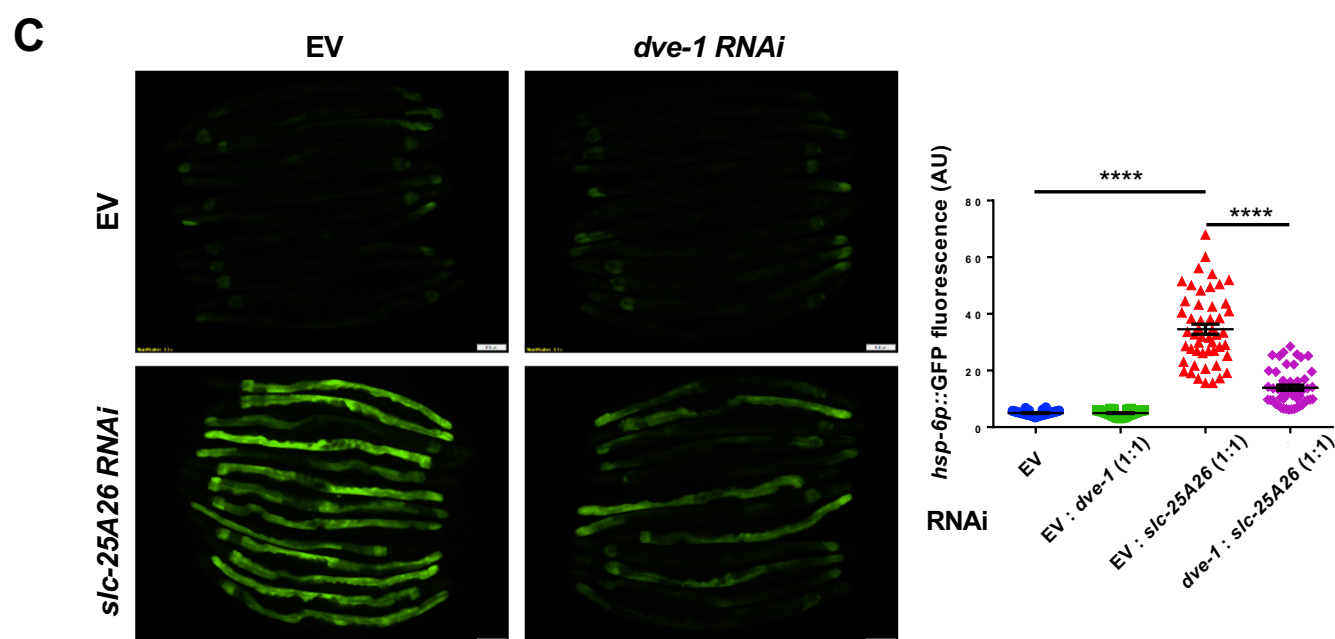

Supplement: Supplementary file 2 — Figure S2. [file ACEL-23-e14103-s005.pdf]

Figure S3

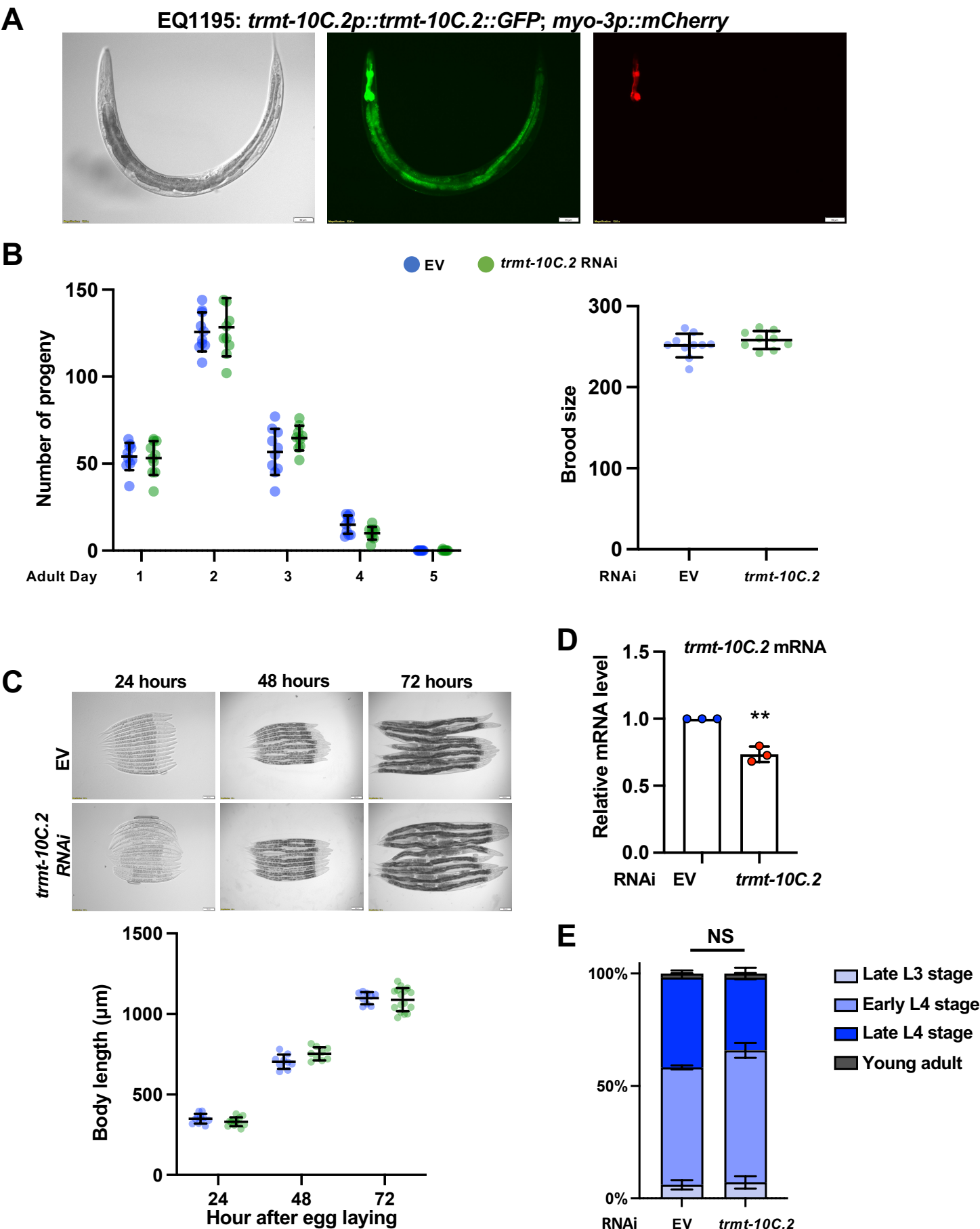

Supplement: Supplementary file 3 — Figure S3. [file ACEL-23-e14103-s004.pdf]

Figure S5

A

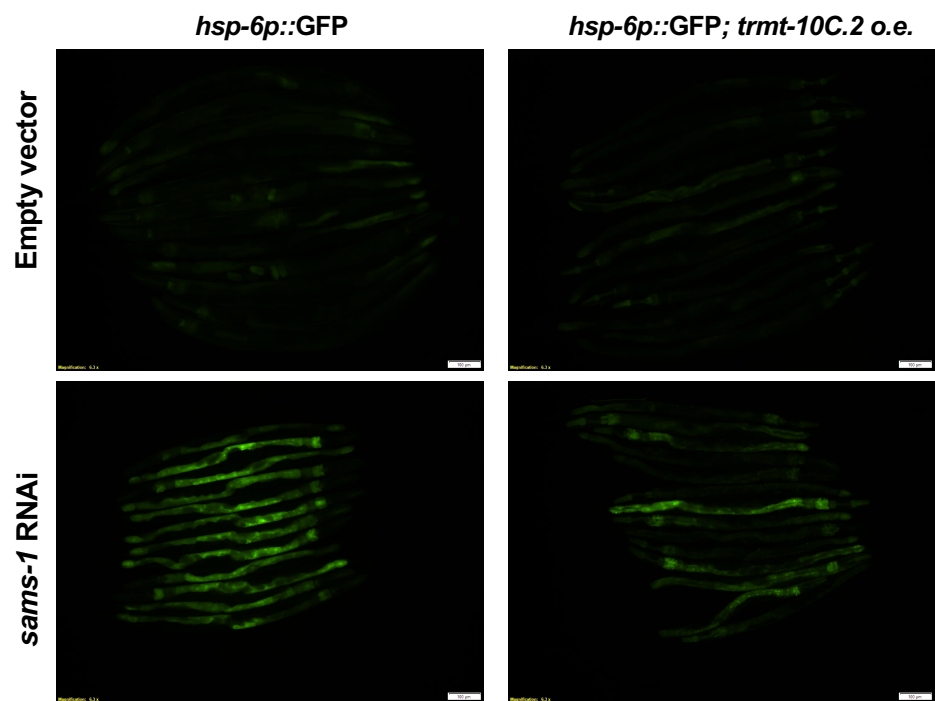

B

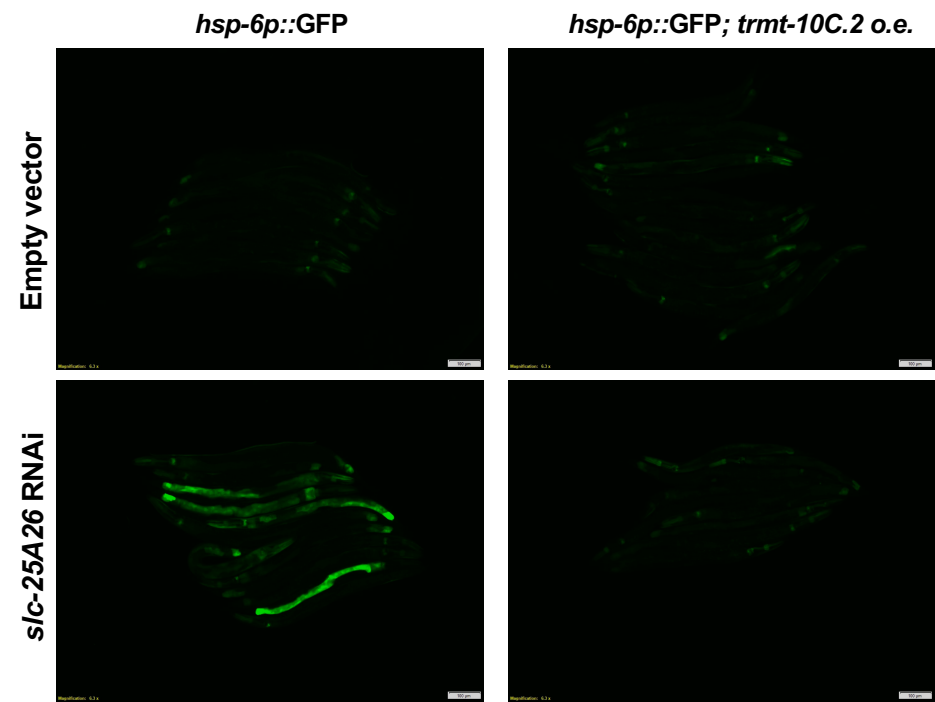

Supplement: Supplementary file 5 — Figure S5. [file ACEL-23-e14103-s001.pdf]

Figure S6

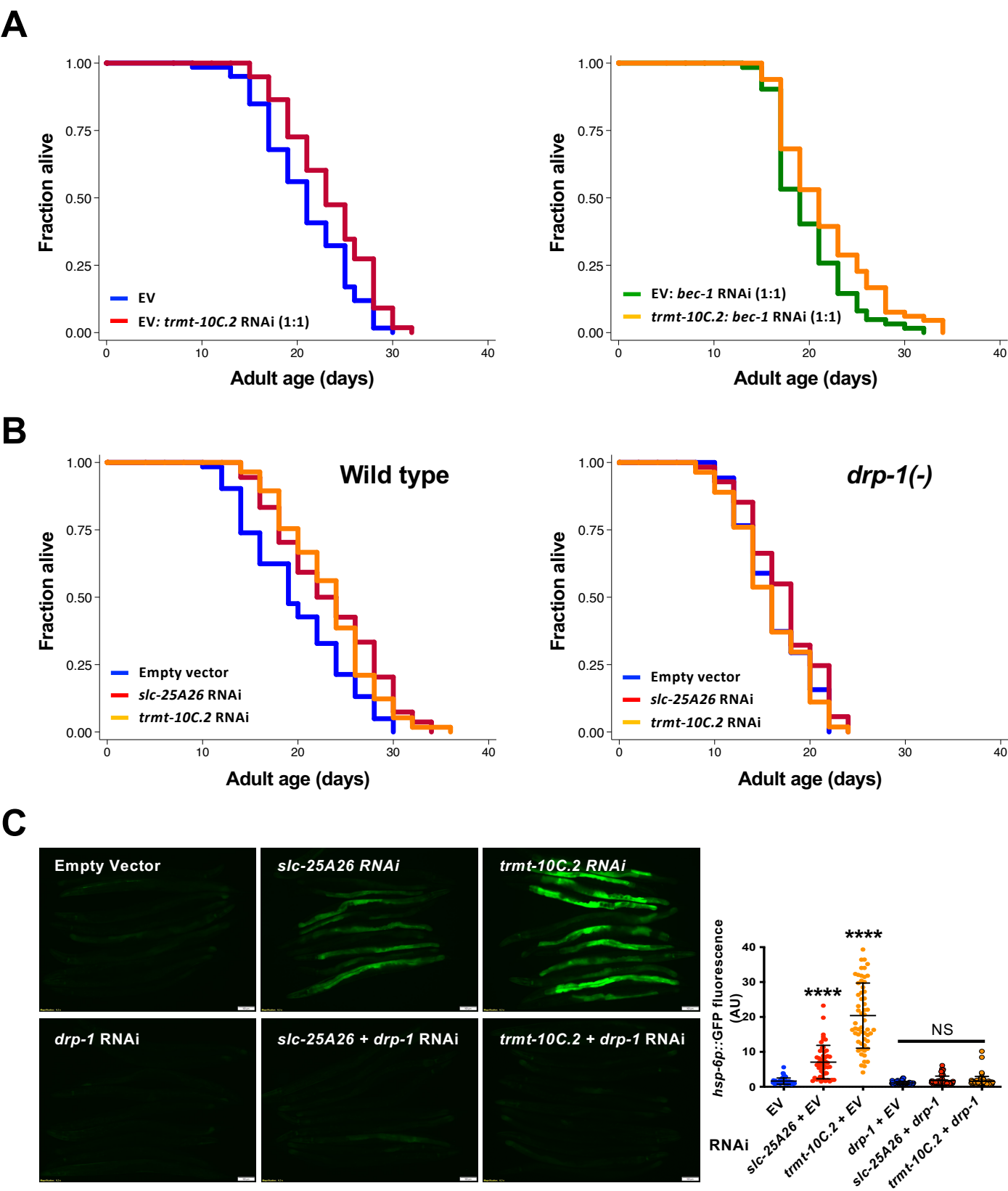

Supplement: Supplementary file 6 — Figure S6. [file ACEL-23-e14103-s007.pdf]
