# Supplementary material for: Mitochondrial S‐adenosylmethionine deficiency induces mitochondrial unfolded protein response and extends lifespan in Caenorhabditis elegans
Source: Aging Cell. 2024 Feb 15;23(4):e14103. doi: 10.1111/acel.14103 (PMC11019128; doi:10.1111/acel.14103)
Supplement: Supplementary file 4 — Figure S4. [file ACEL-23-e14103-s006.pdf]

Figure S4

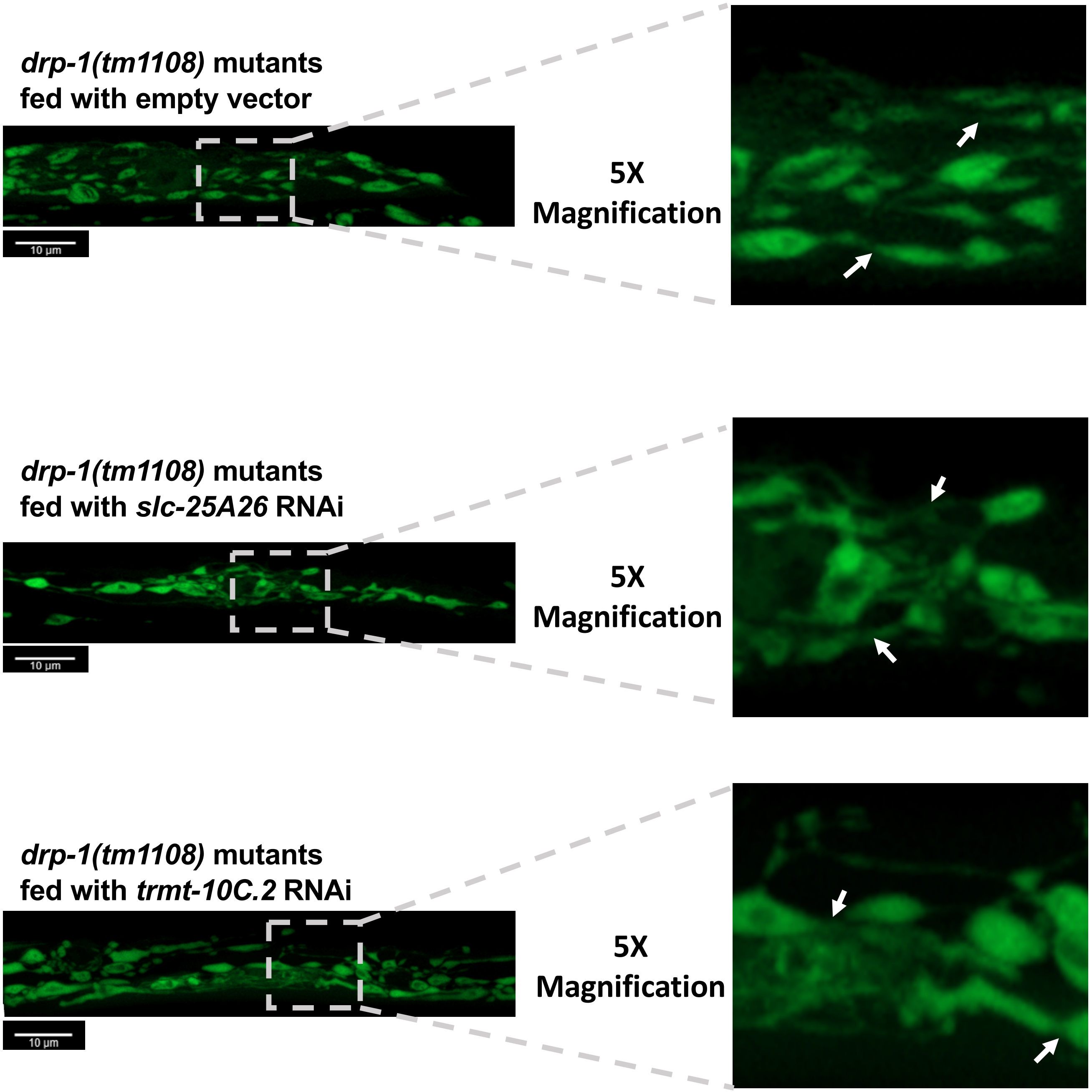
drp-1(tm1108) mutants
fed with empty vector
10 µm
5X
Magnification
drp-1(tm1108) mutants
fed with *slc-25A26* RNAi
10 µm
5X
Magnification
drp-1(tm1108) mutants
fed with *trmt-10C.2* RNAi
10 µm
5X
Magnification
