# Supplementary material for: Mitochondrial S‐adenosylmethionine deficiency induces mitochondrial unfolded protein response and extends lifespan in Caenorhabditis elegans
Source: Aging Cell. 2024 Feb 15;23(4):e14103. doi: 10.1111/acel.14103 (PMC11019128; doi:10.1111/acel.14103)
Supplement: Supplementary file 7 — Table S1. [file ACEL-23-e14103-s002.pdf]

Table S1 | Statistical data for *C. elegans* lifespan experiments

strain name	treatment	mean(days)	*p value*	75%	n	
N2 (WT)	EV	18.1		23	65/72	Fig. 6A
N2 (WT)	*slc-25A26* RNAi	20.5	0.0061	23	62/72	
N2 (WT)	EV	18.0		23	65/72	
N2 (WT)	*slc-25A26* RNAi	20.1	0.0099	23	61/72	
N2 (WT)	EV	18.0		21	79/84	Fig. 6B
N2 (WT)	*trmt-10C.2* RNAi	21.3	0.0019	25	81/84	
N2 (WT)	EV	19.2		24	58/84	
N2 (WT)	*trmt-10C.2* RNAi	21.6	0.0174	24	71/84	
N2 (WT)	EV	19		21	68/72	Fig. 6C
N2 (WT)	*slc-25A26* RNAi	21.1	0.0029 (vs N2)	24	60/72	
sams-1(-)	EV	24.3		31	62/72	
sams-1(-)	*slc-25A26* RNAi	24.2	0.8948 (vs *sams-1*)	38	65/72	
N2 (WT)	EV	17.1		22	58/72	Fig. 6D
N2 (WT)	*sams-1* RNAi	23.5	<0.0001	22	61/72	
trmt-10C.2 oe	EV	15.6		28	65/84	
trmt-10C.2 oe	*sams-1* RNAi	19.3	0.0002	20	76/84	
N2 (WT)	EV	17.5		22	59/71	
N2 (WT)	*sams-1* RNAi	21.8	<0.0001	22	50/71	
trmt-10C.2 oe	EV	15.2		28	48/70	
trmt-10C.2 oe	*sams-1* RNAi	17.1	0.0164	20	54/69	
N2 (WT)	EV	17.9		21	77/84	Fig. 6E
N2 (WT)	*ubl-5* RNAi	16.8	0.0628 (vs N2)	19	78/84	
sams-1(-)	EV	23.2		29	73/84	
sams-1(-)	*ubl-5* RNAi	17.3	<0.0001 (vs *sams-1*)	21	83/84	
N2 (WT)	EV	18.9		21	78/84	
N2 (WT)	*ubl-5* RNAi	17.1	0.003 (vs N2)	19	74/84	
sams-1(-)	EV	23.5		25	80/84	
sams-1(-)	*ubl-5* RNAi	18.4	<0.0001 (vs *sams-1*)	19	83/84	
N2 (WT)	EV	18.6		22	56/84	Fig. 6F
N2 (WT)	*dve-1* RNAi	13.1	<0.0001 (vs N2)	14	67/84	
sams-1(-)	EV	24.3		29	62/84	
sams-1(-)	*dve-1* RNAi	13	<0.0001 (vs *sams-1*)	14	66/84	
N2 (WT)	EV	17.6		22	61/72	
N2 (WT)	*dve-1* RNAi	13	<0.0001 (vs N2)	15	49/72	
sams-1(-)	EV	22.7		27	43/72	
sams-1(-)	*dve-1* RNAi	14.1	<0.0001 (vs *sams-1*)	15	63/72	
N2 (WT)	EV	20.9		23	59/70	Fig. S6A
N2 (WT)	*trmt-10C.2* RNAi	23.3	0.0062 (vs EV)	25	56/71	
N2 (WT)	*bec-1* RNAi	19.7	0.114 (vs EV)	21	62/72	
N2 (WT)	*trmt-1:bec-1* RNAi	21.6	0.0147 (vs *bec-1*)	23	66/71	
N2 (WT)	EV	19.9		24	61/72	Fig. S6B
N2 (WT)	*slc-25A26* RNAi	23.3	0.0014 (vs N2 EV)	28	54/72	
N2 (WT)	*trmt-10C.2* RNAi	23.3	0.004 (vs N2 EV)	26	57/72	
drp-1(-)	EV	16.2		20	51/72	
drp-1(-)	*slc-25A26* RNAi	17.2	0.131 (vs *drp-1(-)* EV)	20	53/72	
drp-1(-)	*trmt-10C.2* RNAi	15.9	0.7241 (vs *drp-1(-)* EV)	20	54/72	
N2 (WT)	EV	20.7		23	64/72	
N2 (WT)	*slc-25A26* RNAi	21.7	0.1012 (vs N2 EV)	26	62/71	
N2 (WT)	*trmt-10C.2* RNAi	22.7	0.0250 (vs N2 EV)	26	67/71	
drp-1(-)	EV	16.4		19	63/72	
drp-1(-)	*slc-25A26* RNAi	16	0.3176 (vs *drp-1(-)* EV)	17	57/72	
drp-1(-)	*trmt-10C.2* RNAi	17.4	0.3001(vs *drp-1(-)* EV)	19	61/72	
