# Supplementary material for: Mitochondrial S‐adenosylmethionine deficiency induces mitochondrial unfolded protein response and extends lifespan in Caenorhabditis elegans
Source: Aging Cell. 2024 Feb 15;23(4):e14103. doi: 10.1111/acel.14103 (PMC11019128; doi:10.1111/acel.14103)
Supplement: Supplementary file 8 — Data S1. [file ACEL-23-e14103-s003.pdf]

Mitochondrial SAM deficiency induces mitochondrial unfolded protein response and longevity in *C. elegans*

Supplementary Figure Legends

Figure S1.

(A) Representative images showing the expression pattern of SLC-25A26 in L4 transgenic worms carrying *slc-25A26p::GFP.* scale bars = 50 µm. **(B)** Efficiency of *slc-25A26* RNAi in wild-type animals. ***, p<0.001, Student's t-test **(C)** Progeny and brood size of wild-type worms grown on the empty vector (EV) and *slc-25A26* RNAi bacteria. Data represent means ± SD for 10 individual worms. **(D)** Representative images and quantification of body length of wild-type worms grown on empty vector (EV) and *slc-25A26* RNAi bacteria from hatching for 24, 48, and 72 hours respectively. **(E)** The developmental stages of wild-type worms grown on empty vector (EV) and *slc-25A26* RNAi bacteria at 56 hours after hatching. **(F)** Semi-quantitative PCR analysis of *sams-1*, *sams-3/4*, and *sams-5* transcripts in L4 N2 worms grown on empty vector or *sams-1* RNAi bacteria. Serial dilutions of 5×, 10× and 20× cDNA were used for the PCR reactions.

Figure S2

(A) Representative images of *hsp-6p::GFP* expression in Day 1 adult worms with indicated genotypes and treatments (left). F_1 progenies of *hsp-6p::GFP* transgenic worms grown on either empty vector (EV) or *sams-1* RNAi bacteria were placed on UV-killed OP50 bacteria supplemented with vehicle, 2 mM methionine, or 2 mM homocysteine. Results are shown as mean ± SD for n = 50 per treatment. Data were analyzed using two-way ANOVA test with Tukey's multiple comparison test. Levels of significance are shown as NS, not significant. Scale bar = 100 µm. **(B)** *hsp-6p::GFP* expression in Day 1 adult worms fed with empty vector, *slc-25A26* RNAi, *atfs-1 RNAi*, or *slc-25A26:atfs-1*(1:1) RNAi bacteria. **(C)** *hsp-6p::GFP* expression in Day 1 adult worms fed with empty vector, *slc-25A26* RNAi, *dve-1* RNAi, or *slc-25A26 :dve-1*(1:1) RNAi bacteria. Right panels, quantitative analysis of *hsp-6p::GFP* expression for **B** and **C.** ****, p<0.0001, two-way ANOVA with Tukey's multiple comparison test.

Figure S3

(A) Representative images showing the expression pattern of TRMT-10C.2 in L4 transgenic worms carrying *trmt-10p::trmt-10C.2::GFP; myo-3p::mCherry*. scale bars = 50 μm. **(B)** Progeny and brood size of wild-type worms grown on empty vector (EV) and *trmt-10C.2* RNAi bacteria. Data represent means ± SD for 10 individual worms. **(C)** Representative images and quantification of body length of wild-type worms grown on empty vector (EV) and *trmt-10C.2* RNAi bacteria from hatching for 24, 48 and 72 hours respectively. **(D)** Efficiency of *trmt-10C.2* RNAi in wild-type animals. **, $p<0.01$, Student's t-test **(E)** The developmental stages of wild-type worms grown on empty vector (EV) and *trmt-10C.2* RNAi bacteria at 56 hours after hatching.

Figure S4

Representative confocal images of the mitochondrial morphology of mtGFP of *myo-3p::GFP(mit); drp-1(tm1108)* worms grown on empty vector, *slc-25A26*, or *trmt-10C.2* RNAi bacteria. Scale bar, 10 μm.

Figure S5

Representative images of **(A)** *hsp-6p::GFP* expression in Day 1 wild-type or *trmt-10C.2*-overexpressing worms grown on empty vector (EV) and *sams-1* RNAi bacteria. **(B)** *hsp-6p::GFP* expression in Day 1 wild-type or *trmt-10C.2*-overexpressing worms fed with empty vector (EV) and *slc-25A26* RNAi bacteria.

Figure S6

(A) Lifespan analysis of wild-type N2 animals treated with empty vector (blue), *trmt-10C.2:*EV (1:1, orange), *bec-1*:EV (1:1, green), or *trmt-10C.2*:*bec-1* (1:1, orange) RNAi bacteria.**(B)** Lifespan analysis of wild-type N2 animals and *drp-1(-)* mutants treated with empty vector (blue), *slc-25A26* (red), or *trmt-10C.2* (orange) RNAi bacteria. Additional lifespan replicates are included in Table S1. **(C)** Representative images of *hsp-6p::GFP* expression in wild-type worms grown on empty vector (EV), *slc-25A26*:EV (1:1), *trmt-10C.2*:EV (1:1), *drp-1*:EV (1:1), *drp-1:slc-25A26*(1:1) and *drp-1:trmt-1(*1:1) RNAi bacteria. Right panel, quantitative analysis of *hsp-6p::GFP* expression. Data were analyzed by two-way ANOVA with Tukey's multiple

comparison test.. Levels of significance were shown as ****, $p<0.0001$; NS, not significant.
